# N6-methyladenosine methyltransferase KIAA1429 promoted ovarian cancer aerobic glycolysis and progression through enhancing ENO1 expression

**DOI:** 10.1186/s13062-023-00420-7

**Published:** 2023-10-09

**Authors:** Lijuan Gan, Shengchao Zhao, Yang Gao, Yuwen Qi, Min Su, Anjin Wang, Hongbing Cai

**Affiliations:** 1 Department of Gynecologic Oncology, Zhongnan Hospital of Wuhan University, Wuhan, People’s Republic of China; 2 Hubei Key Laboratory of Tumor Biological Behaviors, Wuhan, People’s Republic of China; 3 Hubei Cancer Clinical Study Center, Wuhan, People’s Republic of China; 4 Center of Interventional Medicine, The Fifth Affiliated Hospital of Sun Yat-sen University, Zhuhai, People’s Republic of China

**Keywords:** Ovarian cancer, KIAA1429, ENO1, N6-methyladenosine, Aerobic glycolysis

## Abstract

**Background:**

Despite improvements in prognosis due to advances in treatment, including surgery, genetic screening, and molecular targeted therapy, the outcomes of ovarian cancer (OC) remain unsatisfactory. Internal mRNA modifications are extremely common in eukaryotes; N6-methyladenosine (m6A) alteration has significant effects on mRNA stability and translation, and it is involved in the pathophysiology of numerous diseases related to cancer.

**Methods:**

Bioinformatics analysis, quantitative real-time polymerase chain reaction and Western blotting were used to detect the expression of vir-like m6A methyltransferase associated (KIAA1429) in OC tissues and cell lines. Several different cell models and animal models were established to determine the role of KIAA1429 in glucose metabolism reprogramming and the underlying molecular mechanism of OC. The mechanism of oncology functional assays, co-immunoprecipitation and a luciferase reporter gene was employed to ascertain how KIAA1429 interacts with important molecular targets.

**Results:**

We reported that KIAA1429 was overexpressed in OC and predicted a poor prognosis. Functionally, KIAA1429 promoted cell growth by inducing proliferation and inhibiting necrosis. Mechanistically, KIAA1429 promoted tumor progression and glycolysis via stabilizing ENO1 mRNA in a way dependent on m6A. Furthermore, we investigated that the SPI1 transcription factor is the main transcription factor that regulates KIAA1429 transcription in OC.

**Conclusion:**

Our findings revealed that SPI1/KIAA1429/ENO1 signaling is a novel molecular axis and raises awareness of the vital functions of the changes in KIAA1429 and m6A changes in the metabolic reprogramming of OC. These results identified new potential biomarkers and treatment targets for OC.

**Supplementary Information:**

The online version contains supplementary material available at 10.1186/s13062-023-00420-7.

## Introduction

Five-year survival rates for ovarian cancer (OC) are less than 45%, making it the eighth leading cause of death from cancer in women worldwide [1]. Although surgery, chemotherapy, and immunotherapy have made significant progress recently, more efforts have to be made to improve the survival rate of OC patients [2, 3]. Most ovarian cancer patients will eventually relapse due to drug resistance [1, 4]. Therefore, new biological tumor marker molecules and theoretical systems are urgently needed to improve the early diagnosis of OC and the development of targeted therapy.

Recently, the study of RNA epitranscriptomics is becoming increasingly popular. Until now, over 150 chemical alterations of RNA have been discovered, including in protein-coding and non-coding RNAs [5]. Among them, N6-methyladenosine (m6A) is the most prevalent internal change in eukaryotic mRNA and is a factor in the stability of cytoplasmic mRNA [6, 7]. For example, Tao Liu et al. reported that the m6A reader YTH N6-methyladenosine RNA binding protein F1 (YTHDF1) enhances the development of OC by boosting eukaryotic initiation factor 3C (EIF3C) translation [8]; Xiao Yang et al. discovered that methyltransferase 14, N6-adenosine-methyltransferase subunit (METTL14) inhibits colorectal cancer proliferation and metastasis by downregulating oncogenic long non-coding RNA X inactive specific transcript (XIST) [9]; Tian Lan et al. reported that KIAA1429 promotes liver cancer progression through GATA [10]. Huilin Huang et al. reported that insulin-like growth factor-2 (IGF-2) mRNA-binding proteins and 3 (IGF2BP1/2/3), as a new family of m6A reading proteins, protect m6A modified mRNA from being destroyed. IGF2BPs preferentially recognized m6A-modified mRNA, and promoted the stability of thousands of potential mRNA targets (including MYC) in a m6A-dependent manner, thus comprehensively affecting gene expression and output [7]. Furthermore, as a m6A code reader, IGF2BPs plays a carcinogenic role in cancer cells, possibly by stabilizing methylated mRNAs of cancer-causing targets, such as MYC [7]. Notably, it is reported that KIAA1429 knockdown results in a median fourfold decrease in m6A peak scores, which is substantially and significantly more prominent than that achieved upon knockdown of either METTL3 or METTL14 [11], suggesting the importance of KIAA1429 in the methyltransferase complex. However, the exact mechanism of vir-like m6A methyltransferase associated (KIAA1429) and the potential regulatory mechanism in OC remain poorly understood.

By means of aerobic glycolysis, commonly known as the Warburg effect, cancer cells preferentially convert glucose to lactate. A recent research study has demonstrated that the Warburg effect is crucial for cancer initiation and development [12, 13]. Disorders of energy metabolism, particularly the Warburg effect, are hallmarks of many cancers, including OC. Large amounts of lipids, proteins, and nucleotides are produced during glycolysis, which helps accelerate cancer cell proliferation and creates an acidic microenvironment required for cell migration and invasion. Enolase1(ENO1) is a glycolytic enzyme that has a crucial function in aerobic glycolysis by converting 2-phosphoglycerate to phosphoenolpyruvate and is a significant contributor to the Warburg effect in various cancers.

In this work, we identified that KIAA1429 was typically and significantly elevated in OC patients, and high levels of KIAA1429 expression were linked to a worse prognosis. KIAA1429, as an oncogene, promotes the proliferation and metastatic capacity of OC. Subsequently, metabolomics and transcriptomics revealed that KIAA1429 promoted Warburg-like phenotypes with enhanced glycolysis and upregulated glucose uptake, lactate production, and extracellular acidification rate in OC cells. Methylated RNA immunoprecipitation (MeRIP) analysis and dual-luciferase reporter assay revealed that KIAA1429 reduced the degradation of ENO1 mRNA in an m6A-dependent way, hence enhancing glycolysis and OC development. Furthermore, we identified SPI1 as the major transcription factor of KIAA1429 in OC. Our report has identified KIAA1429 as a novel oncogenic molecule in OC and indicates that the SPI1/KIAA1429/ENO1 signaling pathway may be a novel mechanism for the formation and development of OC.

## Materials and methods

### Clinical specimens and cell culture

Epithelial ovarian cancer (EOC) tissue samples and normal ovarian tissues were acquired from surgical specimens of patients between May 2019 and June 2022 at Wuhan University Zhongnan Hospital. This research study was authorized by the Wuhan University Zhongnan Hospital Ethics Committee (KELUN2021087) according to the principles outlined in the Declaration of Helsinki. The hospital pathology service validated the pathologies in the EOC tissue samples. Shanghai Zeye Bioengineering (Shanghai, China), and Procell Life Science & Technology (Wuhan, China), were all used to obtain OC cell lines ES-2, Hey, OVCAR-3, SKOV3, A2780, and the human SV40-transformed immortal cell line IOSE80. ES-2, Hey, OVCAR-3, SKOV3, and A2780 cells were cultured in DMEM (Life Technologies, Carlsbad, CA, USA) supplemented with 10% fetal bovine serum (FBS; BI, Kibbutz, Israel). IOSE80 cells were grown in 10% FBS RPMI1640 (Life Technologies). These cells were cultured in a 37 °C incubator with 5% CO_2_. Cell lines were validated by STR profiling, and there was no mycoplasma contamination.

### RNA isolation and quantitative real-time PCR (qRT–PCR)

Total RNA was isolated from ES-2, Hey, OVCAR-3, SK-OV-3, IOSE80, and A2780 cell lines and tissues using TRI reagent (Invitrogen, CA, USA). We used a DNA reverse transcription kit and followed the manufacturer's instructions to perform reverse transcription (Vazyme, Nanjing, China). qRT–PCR was carried out utilizing SYBR Master Mix (Vazyme). The primers were manufactured by Sangon Biotech (Shanghai, China). The sequences of the qRT–PCR primers utilized in this work are included in Additional file 1: Table S1.

### Western blot analysis

Western blot analysis was performed as previously described [13]. Additional file 1: Table S2 contains a list of antibodies utilized in this work.

### Immunohistochemical staining

Fixation, embedding, and sectioning of tissues. The paraffin-embedded tissues were deparaffinized and rehydrated with xylene and ethanol. The slides were blocked for 20 min in Tris-ethylenediaminetetraacetic acid solution to retrieve antigens. Consequently, slides were incubated in H_2_O_2_-methanol for 10 min. Approximately one hour was spent blocking the slides with 4% donkey serum/ TBST. After incubating the slides with the primary antibody for 60 min at room temperature, they were treated with the biotinylated secondary antibody for an additional overnight at 4 °C. The slides were dehydrated (70% ethanol-xylene replacement) and then mounted with Gum Arabic (Sigma-Aldrich, Oakville, ON, Canada).

### Cell transfection

Plasmids, siRNA, and shRNA were purchased from Wuhan Jinkairui biological engineering co. (Wuhan, China). We cotransfected 293 T cells with an shRNA packaging plasmid to produce lentiviral particles carrying the shRNA (pCMVΔdR8.91, 1.0 µg). After 48 h of transfection, viral supernatants were collected, centrifuged to separate the components, and then filtered to remove any lingering viruses (0.45 µM). Puromycin (2 g/mL) was used to select cells with stable expression. GenMuteTM siRNA transfection reagent (SignaGen Laboratories, MD, USA) was utilized for siRNA transfection, and the procedure was conducted per the manual guidelines. Additional file 1: Table S3 contains the sequences of the shRNA and siRNA tested in this investigation.

### Cell counting kit-8 (CCK-8) assay and colony formation assay

CCK-8 was purchased from MedChemExpress (Shanghai, China) to detect cell viability. Each group of cells was suspended in 96-well plates at a density of 2000 cells/well. Culture medium was removed at the time point of 0 h, 24 h, 48 h, 72 h, and 96 h, respectively, and the cells were incubated with 100 μl complete culture medium containing 10 μl cell counting kit-8 (CCK-8, Shanghai, China) reagent for 1 h. The cell proliferative ability was determined by measuring the optical density at 450 nm. The colony-forming assay was utilized to identify the creation of cell colonies. Briefly, 1000 cells were counted in six-well plates and grown for two weeks. Subsequently, cells were fixed with 4% paraformaldehyde for 20 min and washed with PBS. Consequently, the cells were stained with 0.2% crystal violet and the count of stained colonies was recorded.

### Apoptosis analysis

The Annexin V-FITC/PI apoptosis detection kit (Lianke, Hangzhou, China) was utilized for apoptosis detection. Briefly, cells were trypsinized by EDTA-trypsin and resuspended in 500 μL of binding buffer. Add FITC Annexin V (5 μL) and 10 μL PI, incubate in the dark for 15 min, and gently vortex the sample to disrupt the cells. Emitted fluorescence was quantified using a CytoFLEX flow cytometer (Beckman Coulter). FlowJo version 10.6.0 was used to analyze the data. (FlowJo, USA).

### Scratch wound-healing motility assay and transwell invasion assay

Scratch wound-healing motility assays and Transwell invasion assays were performed as previously described [14].

### In vivo* experiments*

All in vivo experiments were authorized by the Animal Ethics and Welfare Committee of Zhongnan Hospital of Wuhan University. Mice were pooled and randomized into experimental groups for in vivo experiments. Female BALB/c nude mice aged 4–6 weeks were purchased from Jiangsu Jicui Yaokang Biotechnology Co. (Jiangsu, China) For tumorigenesis assay, we establish a xenograft tumor model by subcutaneous injection of OVCAR-3 tumor cells (2 × 10^6^ in Matrigel). Two perpendicular diameters were measured to monitor tumor size every 5 days and nude mice were humanely sacrificed when the mice showed obvious discomfort or the average tumor volume [length × width × width)/2] of the control group exceeded 2000 mm^3^, and all tumors were collected at the same time, and the subcutaneous tumors were removed, weighed, and their sizes were measured. For the lung metastasis model, 1 × 10^6^ OVCAR-3-Luc cells were injected into the tail vein of each mouse. When any mouse showed obvious discomfort or lost more than 20% of its body weight, the experiment was terminated and all mice were sacrificed for the collection of lung. The metastatic nodules were calculated after staining and IHC analysis. For the intraperitoneal metastasis model, 2 × 10^6^ OVCAR-3-Luc cells were injected intraperitoneally into the mice. When any mouse showed obvious discomfort or weight loss of more than 20% or obvious ascites, the experiment was terminated and all mice were sacrificed and the numbers of metastatic nodules were counted. IVIS Spectrum imaging technology was utilized to perform optical imaging (IVIS; Caliper Life Sciences).

### RNA-seq

As input material, 2 µg of RNA per sample was used for RNA samples. Using a spectrophotometer, determine the RNA purity. An RNA concentration was measured using a Qubit. When building sequencing libraries, we followed the manufacturer's recommendations and used the VAHTS mRNA-seq v2 Library Prep Kit for Illumina, and an index code was applied to each sample attribute sequence. Sequencing The libraries were sequenced according to the manufacturer guidelines on the Illumina NovaSeq platform to generate 150 bp paired-end reads. Differentially expressed genes are those with an adjusted *P* value ≤ 0.05 and a fold change ≥ 1.

### Glucose consumption and lactate production

Glucose consumption was measured by the glucose uptake assay following the manufacturer's instructions (ab136955, Abcam, USA). After treatment with 2-DG, cells were placed in a 37 °C incubator for 20 min, followed by cell lysis with extraction buffer and heating at 85 °C for 40 min. Add reaction mix A and incubate at 37 °C for 1 h; add extraction buffer and heat to 90 °C for 40 min; add reaction mix B and read using a microplate reader. The L-lactate test kit was utilized to measure lactate levels (ab65331, Abcam).

### Measurement of extracellular acidification rates (ECAR)

For this study, we used an XFe96 extracellular flux analyzer to evaluate OC cell ECAR (Agilent Technologies). 1 × 10^4^ OC cells were seeded in a Seahorse XFe96 cell culture microplate. The rate of extracellular acidification was analyzed following subsequent injections of oligomycin, carbonyl cyanide-p-trifluoromethoxyphenylhydrazone (FCCP; 1 μM), and a rotenone/antimycin mixture (0.5 μM).

### Dual-luciferase assay

The Dual-Luciferase Test Kit was utilized to conduct the luciferase reporter gene assay (Beyotime Biotechnology, Shanghai, China). The wild-type or mutant KIAA1429 was cloned into the pGL3 plasmid. Lipofectamine 3000 (Thermo Fisher) was used for transient transfections according to the manufacturer's guidelines. After 24 h, we used the Dual-Glo Luciferase Assay System (Promega, USA) to quantify luciferase activity, which we then standardized to firefly luciferase activity using the following formula: firefly luciferase activity/renilla luciferase activity.

### ChIP assay

Briefly, formaldehyde was utilized to cross-link the cells, and then they were quenched with glycine and sonicated in cold water. Anti-KIAA1429 or anti-IgG antibodies were added to the mixture and incubated at 4 °C with rotation for 12 h before being replaced by protein A/G agarose for 6 h. Elution buffer (1% SDS and 0.1 M NaHCO_3_) was used to elute the mixture at 37 °C for 30 min. The mixture was then reversed cross-linking with high salt buffer for 4 h at 65 °C. Quantification was conducted by qRT-PCR utilizing primers that contain the KIAA1429 binding site.

### Statistical analyses

All statistical analyses were performed utilizing Prism version 9.3.1. Utilizing the student's t-test, data from two groups were compared. A two-way repeated-measures ANOVA analyzed the CCK-8 data. To determine the correlations, the Spearman rank coefficient was calculated. Each test was conducted three times in triplicate. In all analyses, a *p* value less than 0.05 was considered to imply statistically significant differences.

## Results

### KIAA1429 was increased in OC and was correlated with a worse prognosis

**Fig. 1:**
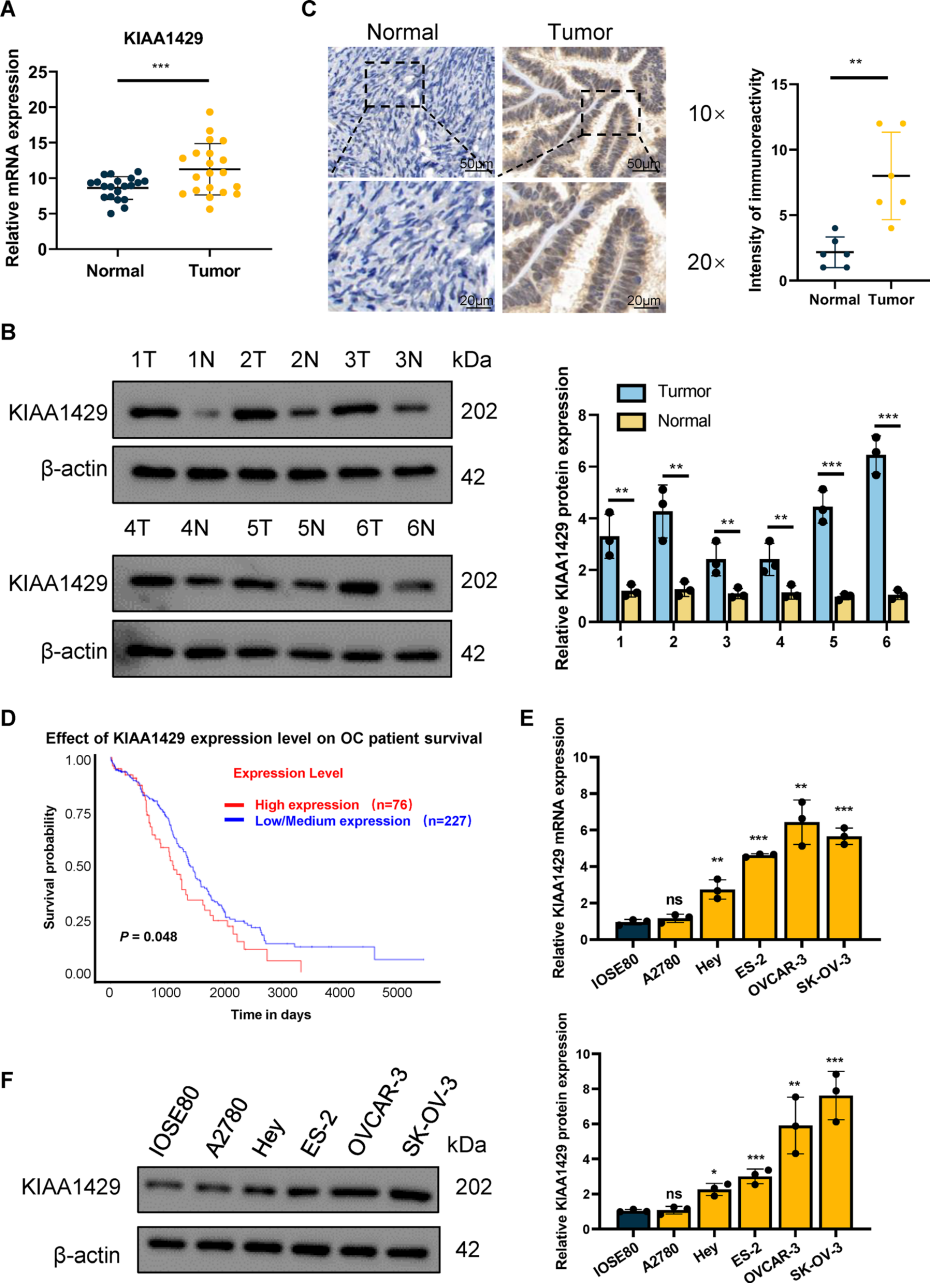
KIAA1429 was highly expressed in OC. A KIAA1429 mRNA expression in normal tissues and EOC tissues. *n* = 20 independent experiments. B KIAA1429 protein expression in normal tissues and EOC tissues. *n* = 6 independent experiments. C Representative immunohistochemical staining of KIAA1429 in normal tissues and EOC tissues (stage III). N: normal tissue; T: tumor tissue. *n* = 6 independent experiments. D Correlations between KIAA1429 mRNA expression and OC prognosis in TCGA database. E, F KIAA1429 mRNA and protein expression in normal ovarian cells and OC cells. *n* = 3 independent experiments; *P < 0.05, **P < 0.01, ***P < 0.001, and ns indicates no significant difference

To evaluate the expression level of KIAA1429 in OC, qRT–PCR, western blotting, and immunohistochemistry were performed on 20 normal ovarian tissues and 20 EOC tissues obtained from the Zhongnan Hospital of Wuhan University. The mRNA expression of KIAA1429 was considerably elevated in EOC tissues compared to normal tissues (Fig. 1A). Additionally, western blotting and immunohistochemical staining further verified that the KIAA1429 protein expression level was higher in EOC tissues than in normal tissues (Fig. 1B, C). Subsequently, we studied the association between KIAA1429 expression and clinical EOC tissue features and found that high KIAA1429 expression was positively correlated with distant metastasis and the federation international of gynecology and obstetrics (FIGO) stage (Additional file 1: Table S4). Kaplan–Meier analysis of the TCGA dataset (http://cancergenome.nih.gov/) revealed that high KIAA1429 expression was related to decreased overall survival (*p* = 0.048; Fig. 1D). Subsequently, we examined the KIAA1429 expression level in different OC cell lines. Compared to the human SV40-transformed immortal cell line IOSE80, KIAA1429 mRNA and protein levels were elevated in OC cell lines (ES-2, Hey, OVCAR-3, SK-OV-3; Fig. 1E, F). These results indicated that KIAA1429 is anticipated to be a potential diagnostic and prognostic biomarker of OC.

### KIAA1429 promoted tumor growth and metastasis

**Fig. 2:**
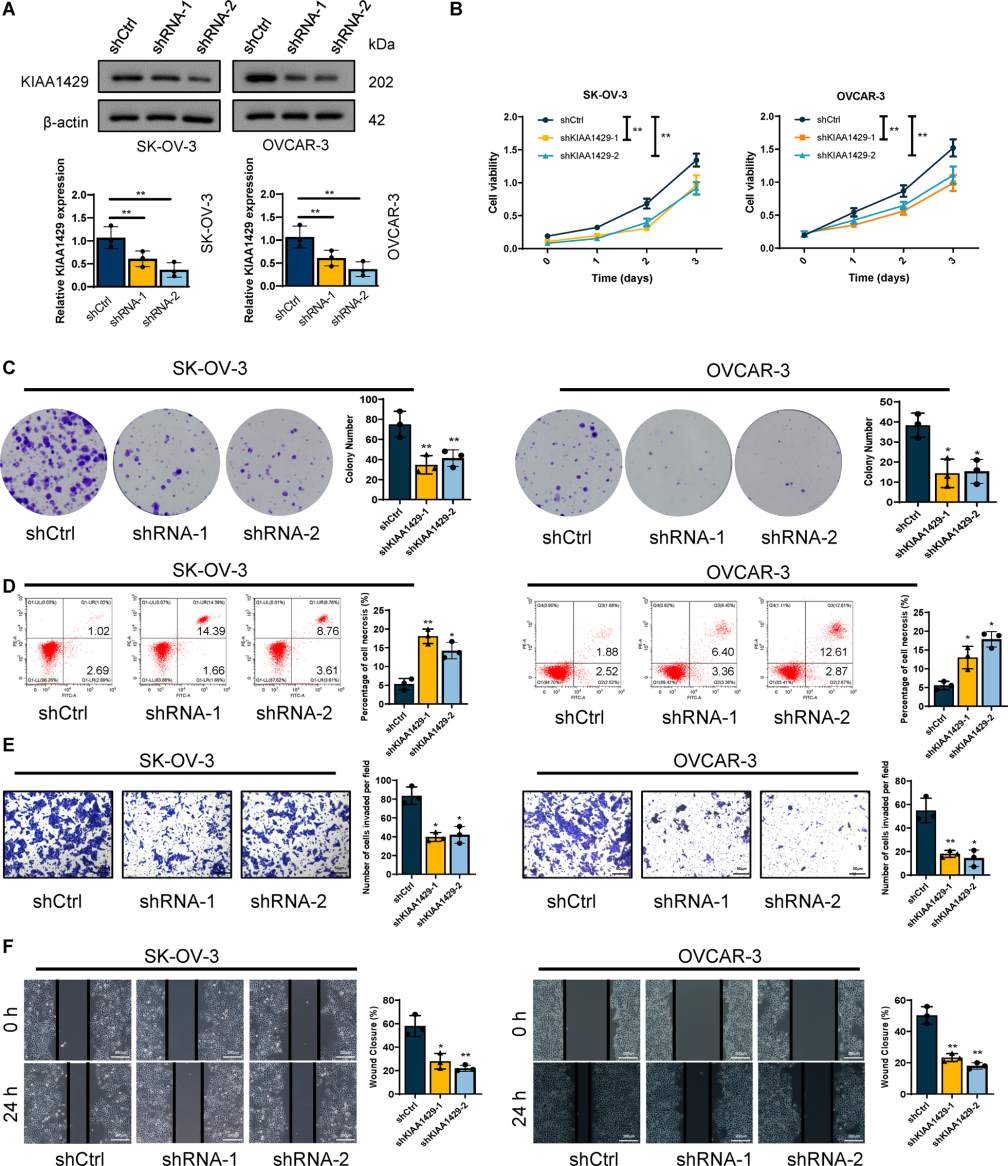
KIAA1429 promoted OC proliferation and metastasis. **A** Western blotting was performed to determine KIAA1429 expression in shRNA control and KIAA1429 knockdown cells.* n* = 3 independent experiments. **B**, **C** Cell proliferation was determined by CCK-8 assay and colony formation assay. *n* = 3 independent experiments. **D** Cell apoptosis were determined by flow cytometry. *n* = 3 independent experiments. **E**, **F** Cell invasion and migration were determined by wound-healing migration assay and Transwell invasion assay. *n* = 3 independent experiments; *P < 0.05, **P < 0.01, and ***P < 0.001

To examine the biological significance of KIAA1429 in OC, we selected SK-OV-3 and OVCAR-3 cell lines with the highest KIAA1429 expression level to construct knockdown models and used A2780 cells with the lowest KIAA1429 expression level to construct an overexpression model. The KIAA1429 knockdown induced by shRNA was examined by qRT-PCR and western blotting (Fig. 2A and Additional file 2: Fig. S1A). We first designed two shRNAs targeting KIAA1429 to establish stable cell lines. The results of CCK-8 and clone formation experiments indicated that KIAA1429 knockdown significantly decreased cell development, while overexpression of KIAA1429 produced the opposite outcome (Fig. 2B, C and Additional file 2: Fig. S1C, D). Moreover, we studied the influence of KIAA1429 on apoptosis/necrosis in OC cell lines. The flow cytometry study indicated that the inactivation of KIAA1429 increased the rate of cell necrosis in OC cell lines (Fig. 2D). Through transwell and scratch assays, we also established that KIAA1429 knockdown markedly repressed the cell invasion and migration, whereas KIAA1429 overexpression showed the opposite effect (Fig. 2E, F, and Additional file 2: Fig. S1E, F). The results of this study indicated that KIAA1429 could increase OC cell proliferation, migration and invasion.

### KIAA1429 knockdown decreased OC cell proliferation and metastasis in nude mice

**Fig. 3:**
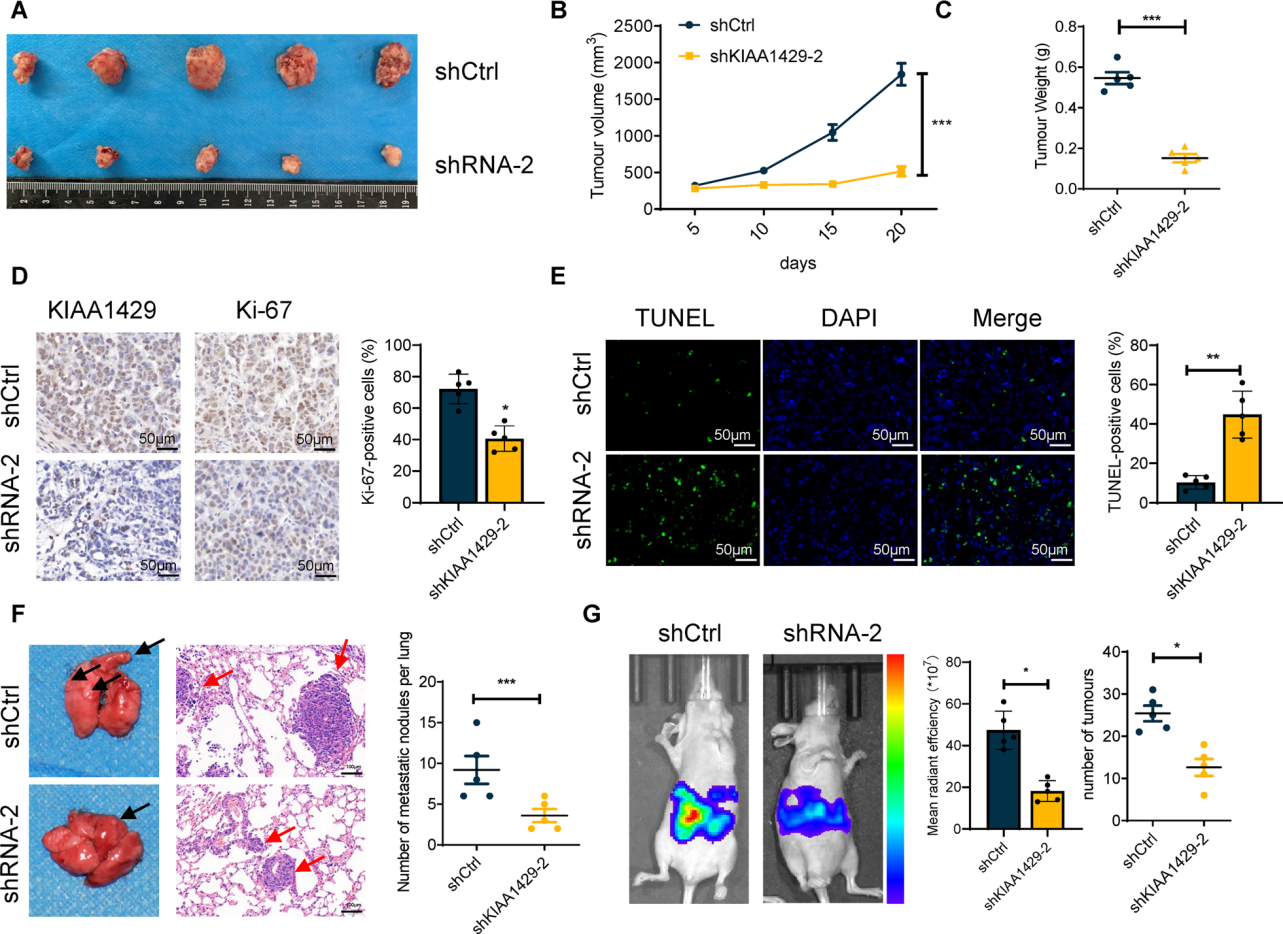
KIAA1429 promoted cell proliferation and metastasis in vivo*.* The Photos taken after subcutaneous tumors removal in BALB/c nude mice after cell inoculation 20 days.* n* = 5 independent experiments. **B** Growth curves of the xenograft tumors in BALB/c nude mice after cell inoculation 20 days.* n* = 5 independent experiments. **C** Weight of xenograft tumors in BALB/c nude mice after cell inoculation 20 days.* n* = 5 independent experiments. **D** IHC staining for Ki-67 and KIAA1429 after cell inoculation 20 days.* n* = 5 independent experiments. **E** Immunofluorescence staining for TUNEL assays after cell inoculation 20 days.* n* = 5 independent experiments. **F** The number of lung metastatic foci in lung metastasis BALB/c nude mice after cell inoculation 40 days are shown. *n* = 5 independent experiments. G The fluorescence intensity values and the number of intraperitoneal metastatic foci in intraperitoneal metastasis BALB/c nude mice after cell inoculation 30 days are shown. *n* = 5 independent experiments; *P < 0.05, **P < 0.01, and ***P < 0.001

We investigated the effects of KIAA1429 knockdown on ovarian cancer (OC) cell proliferation and metastasis in vivo, considering that KIAA1429 knockdown suppresses OC cell proliferation and metastasis in vitro. First, we engrafted shCtrl or shKIAA1429-2, stably transfected OVCAR-3 cells subcutaneously into nude mice. The results of tumor volume and tumor growth curves suggested that tumor growth was significantly slower in the shKIAA1429-2 group compared to the shCtrl group (Fig. 3A, B). Furthermore, the shKIAA1429-2 group formed a lighter tumor weight than the shCtrl group (Fig. 3C). Immunohistochemical staining of xenograft tumor tissues revealed that expression of KIAA1429 was significantly reduced in shKIAA1429-2 group in contrast to the shCtrl group (Fig. 3D), indicating that the KIAA1429 knockdown exerted an inhibitory effect on tumor growth. Furthermore, Ki-67 staining and TUNEL staining of xenograft tumor tissues indicated that KIAA1429 knockdown reduced the number of proliferative cells while increasing the number of apoptotic cells (Fig. 3D, E). Metastasis of ovarian cancer can occur through the coelom, hematogenous or lymphatic routes. Among them, distant lung metastases and direct intraperitoneal dissemination are considered more common. Therefore, models of lung metastasis and peritoneal metastasis were established to validate KIAA1429 in tumor metastases. Models of lung metastasis demonstrated that KIAA1429 knockdown significantly reduced the count of metastatic surface lung nodules (Fig. 3F). The intraperitoneal metastasis experiment further demonstrated that both the number and fluorescence intensity of metastatic foci and intraperitoneal metastatic nodules were significantly diminished after the KIAA1429 knockdown (Fig. 3G).

### KIAA1429 promoted aerobic glycolysis in OC cells

**Fig. 4:**
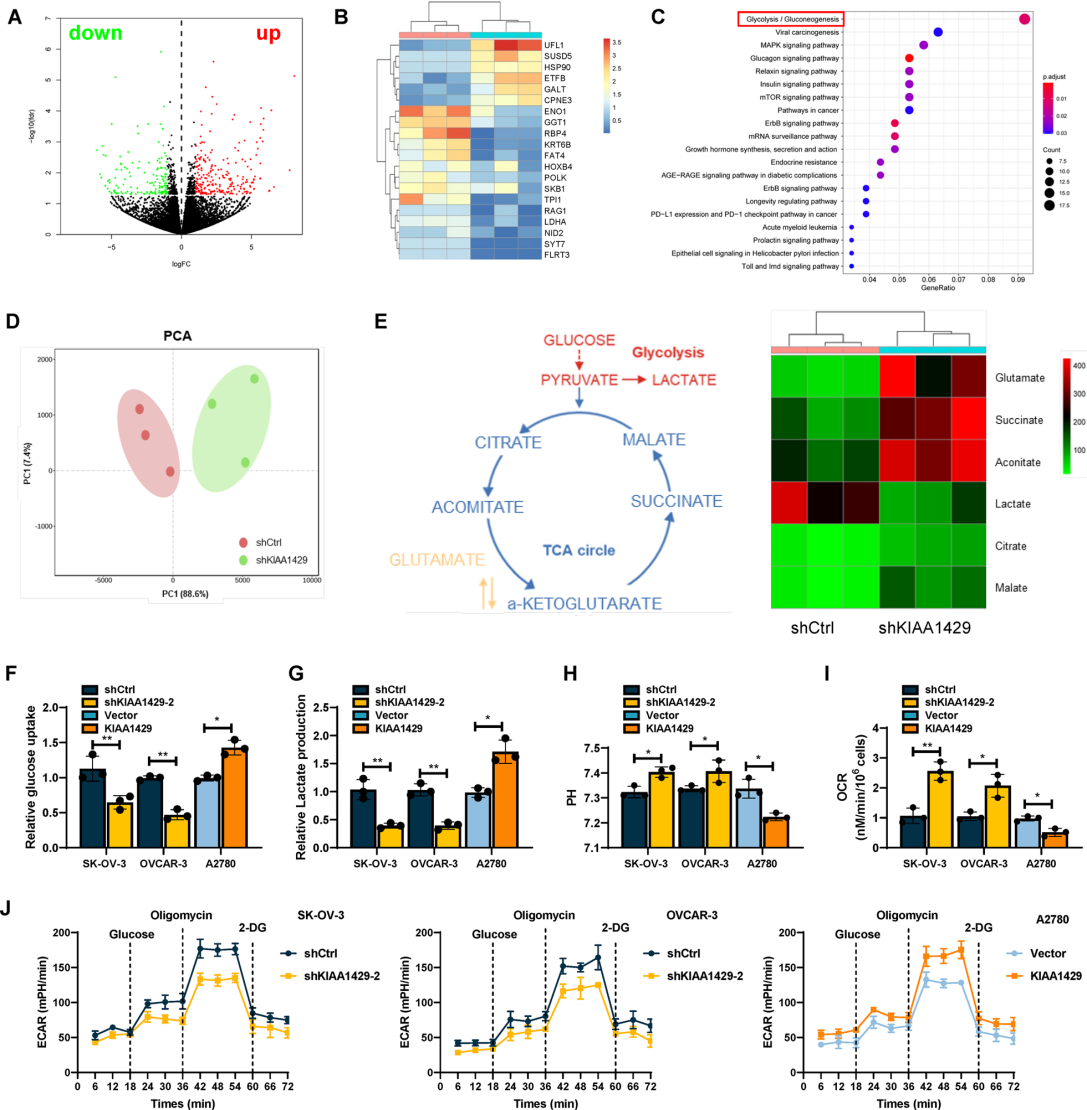
KIAA1429 enhanced aerobic glycolysis on OC cells. **A** Volcano plot showed the differential genes for RNA-seq. *n* = 3 independent experiments. **B** Heatmap showed the 20 most significantly different genes in RNA-seq. *n* = 3 independent experiments. **C** KEGG pathway analysis of RNA-seq data. **D** PCA analysis showed differences between groups. *n* = 3 independent experiments. **E** Nontargeted metabolomics GC–MS analysis of glycolysis and TCA cycle metabolites in OC cells. Heatmap showed changes in metabolites of glycolysis or OXPHOS. *n* = 3 independent experiments. **F**–**I** Glucose uptake (**F**), lactate production (**G**), pH of the culture medium (**H**) and OCR (**I**) were tested in three different cell lines. J The ECAR was measured in three different cell lines using an XF Extracellular Flux Analyzer. *n* = 3 independent experiments; *P < 0.05, **P < 0.01, and ***P < 0.001, and ns indicates no significant difference

To further investigate the possible molecular mechanism by which KIAA1429 impacts OC growth and metastasis, we carried out RNA-seq analysis on OC cell lines infected with shKIAA1429-2 or shCtrl. Volcano plots preliminarily showed the significance of differential gene expression (DEG; Fig. 4A). Gene expression heatmap showed the top ten upregulated and ten downregulated genes in the integrated DEGs between two samples (Fig. 4B). The KEGG enrichment analysis indicated that the glycolysis pathway achieved statistically significant enrichment (Fig. 4C). Altered aerobic glycolysis is a well-established feature of cancer cell energy metabolism. Even in the presence of sufficient oxygen, most tumor cells produce large amounts of energy through hyperglycolytic metabolism, and OC is no exception [15]. We conducted an galactose experiment. We cultured OC cells in galactose and CCK-8 results showed that the proliferation capacity of ovarian cancer cells (SK-OV-3 and OVCAR-3) was significantly reduced in the galactose medium. This indicates that glycolysis is important in OC tumor development (Additional file 7: Fig. S6B). This also suggests that glycolysis plays an important role in the development of ovarian cancer. To complement transcriptome analysis, we conducted an unbiased assessment of the tumor cell metabolome via untargeted metabolomics. The principal component analysis (PCA) showed the relatedness of the samples (Fig. 4D). Heatmap showed differentially expressed metabolites of glucose metabolism pathways (Fig. 4E). To systematically explore whether KIAA1429 influences the Warburg effect of OC cells, we evaluated glucose absorption and lactate generation following KIAA142 knockdown. KIAA1429 knockdown significantly decreased glucose absorption and lactate generation, whereas KIAA1429 overexpression caused the opposite result (Fig. 4F, G). Moreover, KIAA1429 knockdown also increased mitochondrial oxygen consumption rate (OCR) and intracellular pH, whereas KIAA1429 overexpression caused the opposite result (Fig. 4H, I). Consequently, we measured the extracellular acidification rate (ECAR) in OC cells, and the findings demonstrated that basal levels of ECAR significantly decreased in OC cell lines after KIAA1429 knockdown, whereas KIAA1429 overexpression caused the opposite changes (Fig. 4J). These results indicated that KIAA1429 promoted aerobic glycolysis of OC cells.

### ENO1 was discovered to be a possible downstream target of KIAA1429

**Fig. 5:**
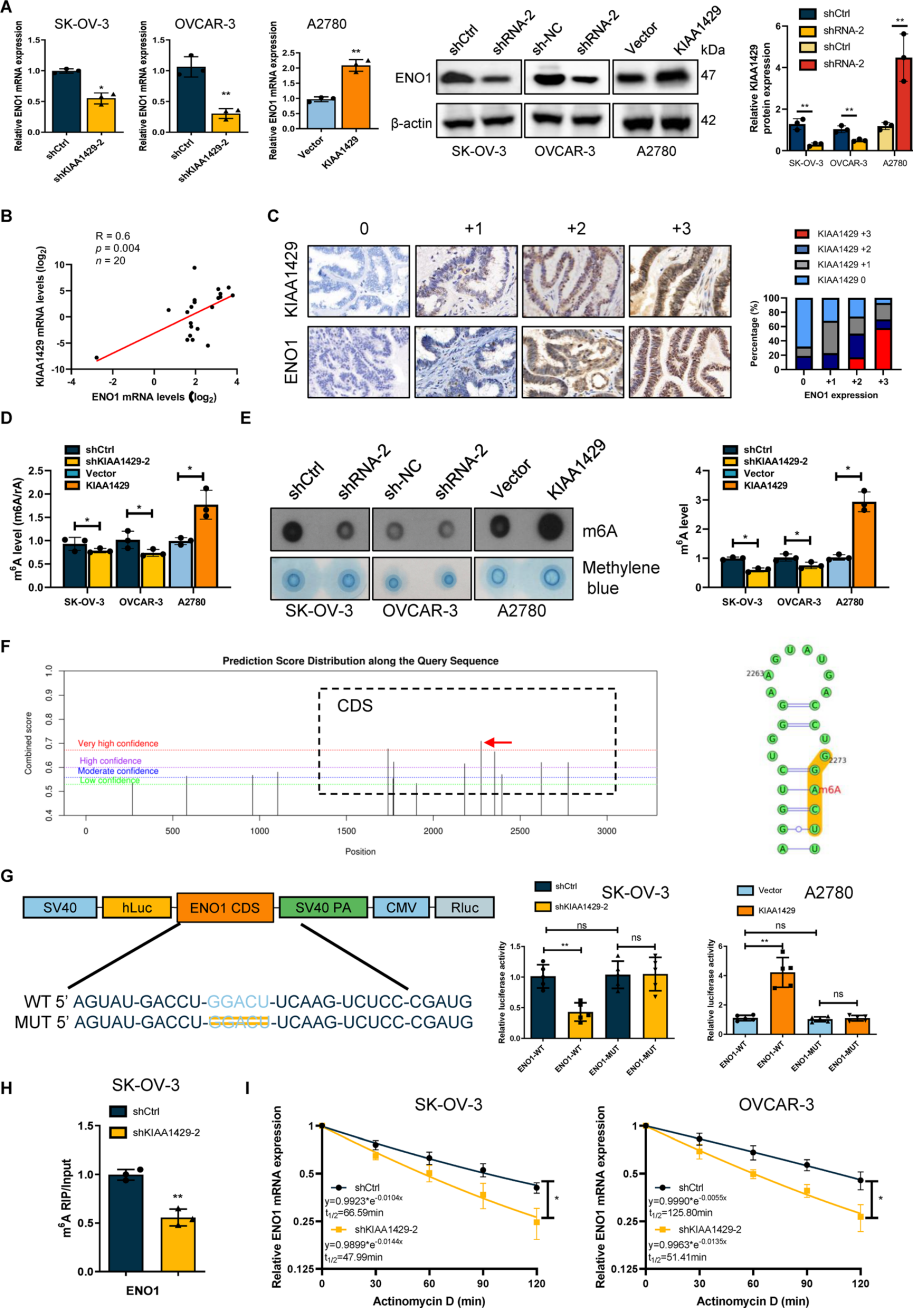
KIAA1429-mediated stabilization of ENO1 mRNA relies on m6A modification. **A** qRT-PCR and Western blotting were performed to determine ENO1 expression in differently treated cells. *n* = 3 independent experiments. **B** Spearman’s rank correlation analyses showed the correlations between KIAA1429 and ENO1. *n* = 20 independent experiments. **C** Correlation between IHC staining intensity for KIAA1429 and ENO1 in tumour sections. *n* = 20 independent experiments. **D** LC/MS results showed the m6A methylation level.* n* = 3 independent experiments. **E** Dot blot showed changes in m6A methylation. Methylene blue staining was used as a control. *n* = 3 independent experiments. **F** The SRAMP database predicted the distribution of m6A sites in ENO1 mRNA. *n* = 3 independent experiments. **G** Luciferase activity was measured and normalized to Renilla luciferase activity. *n* = 3 independent experiments. **H** MeRip-qPCR analysis of m6A levels in ENO1 mRNA. *n* = 3 independent experiments. **I** Changes in ENO1 mRNA stability after ACT-d treatment. *n* = 3 independent experiments; *P < 0.05, **P < 0.01, and ***P < 0.001

We validated our RNA-seq findings with qRT–PCR and western blot, and the findings demonstrated that ENO1 was significantly decreased after KIAA1429 knockdown and increased significantly after overexpression (Fig. 5A, B). Furthermore, we analyzed ENO1 as a possible target for the downstream effects of KIAA1429. We first examined the mRNA and protein levels of KIAA1429 and ENO1 in OC tissue samples by qRT–PCR and immunohistochemical analysis. There was a positive relationship between mRNA levels and protein levels of KIAA1429 and ENO1 (Fig. 5B, C). According to the methyltransferase profile of KIAA1429, we hypothesized that KIAA1429 regulates ENO1 in an m6A-dependent manner. We first examined whether KIAA1429 regulates m6A methylation in OC cells. Liquid chromatography-tandem mass spectrometry (LC/MS) and dot blot analysis presented that the KIAA1429 knockdown significantly decreased m6A levels in OC, whereas overexpression did the opposite (Fig. 5D, E). To understand the m6A modification role in ENO1 mRNA regulation, the methylation site of ENO1 was predicted using the SRAMP database. The prediction results indicated that the CDS region 2273–2278 sites of ENO1 include an obvious m6A site (Fig. 5F). Consequently, we altered ENO1's m6A-preferred locations (GGACU) to construct mutant luciferase plasmids. We selected ENO1 wild-type (WT) or mutant (MUT) luciferase reporter plasmid for luciferase reporter assays. As expected, the luciferase reporter experiment revealed that the WT group's luciferase activity was considerably reduced following KIAA1429 silencing but not the MUT group's luciferase activity (Fig. 5G). Importantly, using methylated RNA immune-precipitation qPCR (Me-RIP qPCR), we observed a significant enrichment of ENO1 mRNA using the m6A antibody, and KIAA1429 knockdown decreased ENO1 mRNA m6A levels (Fig. 5H). After inhibiting RNA synthesis with actinomycin D, we subsequently evaluated ENO1 mRNA expression levels. The results showed that silencing KIAA1429 significantly reduced ENO1 mRNA stability (Fig. 5I). In addition, we overexpressed ENO1-WT plasmid and ENO1-Mut plasmid in the cell lines with ENO1 knockdown, respectively. ENO1-Mut plasmid has no m6A site that can be recognized by KIAA1429. KIAA1429 knockdown can only affect the stability of ENO1 mRNA in cells transfected with ENO1-WT plasmid, but could not affect the stability of ENO1 mRNA in cells transfected with ENO1-Mut plasmid, which proves that KIAA1429' s regulation of ENO1 mRNA stability is m6A-dependent (Additional file 3: Fig. S2A). These data demonstrated that the m6A methyltransferase activity function of KIAA1429 is required for KIAA1429 to increase ENO1 mRNA stability.

### ENO1 promoted tumor growth and metastasis

ENO1 has been demonstrated to enhance the progression of gastric cancer, lung cancer, and breast cancer. However, its significance in OC cells has not been shown. Using siRNA, we generated an ENO1 knockdown OC cell model (Additional file 4: Fig. S3A). CCK-8 and clone creation experiments demonstrated that ENO1 knockdown decreased the proliferative capacity of OC cells (Additional file 4: Fig. S3B, C). Furthermore, wound healing studies and transwell migration studies demonstrated that ENO1 knockdown significantly inhibited the invasive capabilities of OV cells (Additional file 4: Fig. S3D, E). Our research demonstrates that ENO1 is a tumor-promoting factor in OC, increasing cancer proliferation and metastasis.

### KIAA1429 promoted OC growth and metastasis through ENO1

**Fig. 6:**
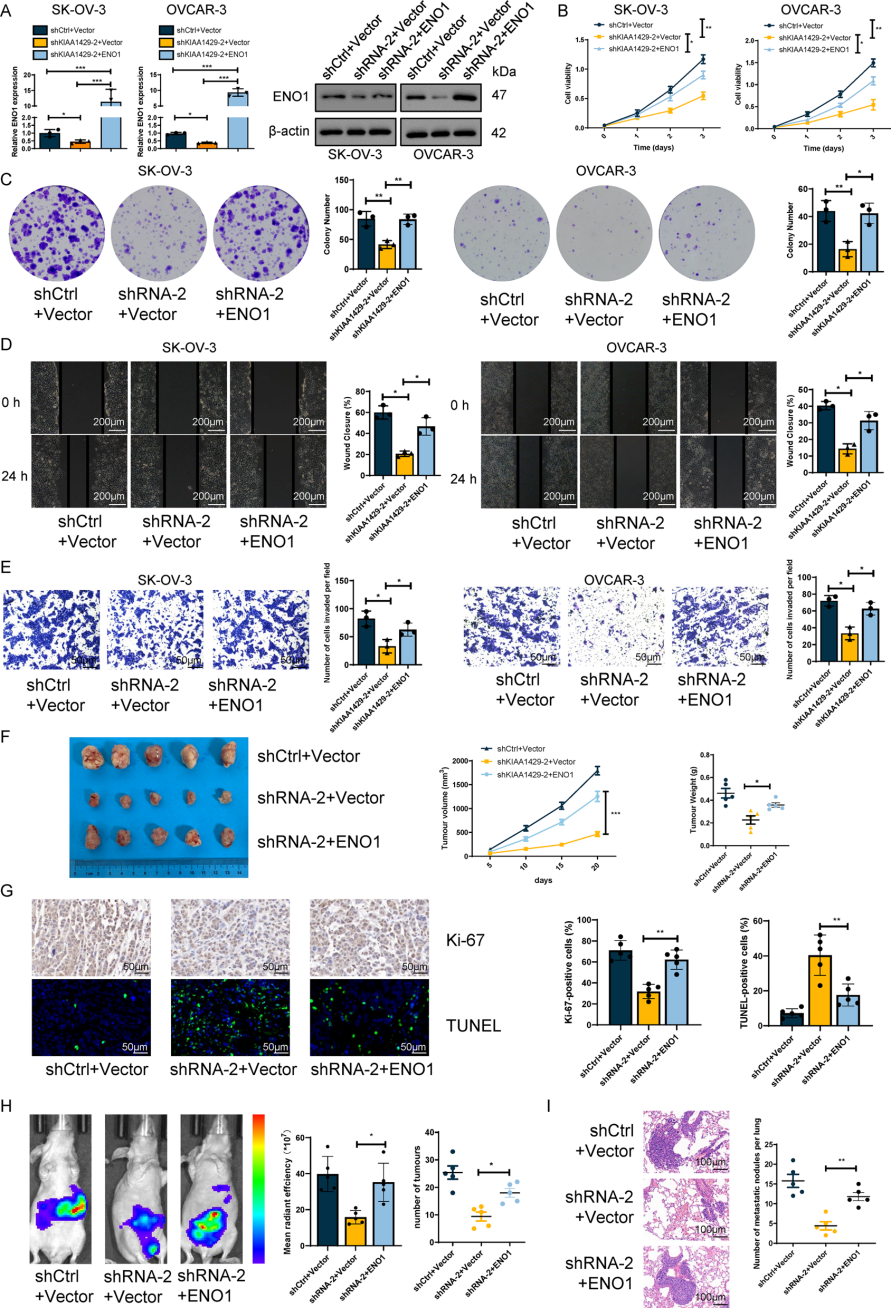
The tumor-promoting function of KIAA1429 depends on ENO1. **A** qRT-PCR and Western blotting were performed to determine ENO1 expression in differently treated cells. *n* = 3 independent experiments. **B**, **C** Cell proliferation was determined by CCK-8 assay and colony formation assay. *n* = 3 independent experiments. **D**, **E** Cell invasion and migration were determined by wound-healing migration assay and Transwell invasion assay. *n* = 3 independent experiments. **F** Curves show the volume and growth of subcutaneous xenografts of OC cells in BALB/c nude mice after cell inoculation for 20 days. *n* = 5 independent experiments. **G** IHC and IF staining for Ki-67 and TUNEL assays in BALB/c nude mice after cell inoculation 20 days. *n* = 5 independent experiments. **H** The fluorescence intensity values and the number of intraperitoneal metastatic foci in intraperitoneal metastasis BALB/c nude mice after cell inoculation for 30 days are shown. *n* = 5 independent experiments. **I** The number of lung metastatic foci in lung metastasis BALB/c nude mice after cell inoculation 40 days are shown. *n* = 5 independent experiments. *P < 0.05, **P < 0.01, and ***P < 0.001

Next, we performed rescue experiments to study the function of ENO1 in KIAA1429-induced cell proliferation and metastasis (Fig. 6A). CCK-8 and cloning assays demonstrated that ENO1 overexpression significantly restored the inhibition of cell proliferation caused by KIAA1429 knockdown (Fig. 6B, C). Moreover, ENO1 overexpression reversed the reduction in invasion and migration ability caused by KIAA1429 silencing (Fig. 6D, E). We then investigated the role of ENO1 in KIAA1429-mediated aerobic glycolysis. The inhibition of glucose uptake and lactate production caused by KIAA1429 knockdown was significantly alleviated by overexpressing ENO1 (Additional file 5: Fig. S4A). Furthermore, ENO1 overexpression significantly reversed the promoting effects of KIAA1429 knockdown on OCR and intracellular pH (Additional file 5: Fig. S4A). The ECAR data revealed that ENO1 overexpression significantly counteracted the suppressive effect of KIAA1429 knockdown on aerobic glycolysis (Additional file 5: Fig. S4B). These results suggested that KIAA1429 exerts aerobic glycolysis through ENO1.

Finally, we performed rescue experiments in vivo. Overexpression of ENO1 counteracted the suppression of KIAA1429 on aggressive characteristics of tumor cells, as shown in an in vivo tumor rescue experiment (Fig. 6F). Furthermore, the inhibitory effect of KIAA1429 knockdown on subcutaneous xenograft tumor proliferation and the promoting effect on cell apoptosis were rescued by ENO1 overexpression (Fig. 6G). Both tail veins injected lung metastasis models, and peritoneal metastasis models demonstrated that ENO1 restored the diminished capacity for abdominal and lung metastases induced by KIAA1429 silencing. (Fig. 6H, I). Our data strongly suggested that ENO1 promotes tumor progression by acting as a downstream target of KIAA1429. In addition, we checked whether KIAA1429 and ENO1 were correlated in other tumor tissues. We checked the TCGA database and demonstrated KIAA1429 and ENO1 were also positive correlated in LIHC and PAAD, suggesting that this correlation was not specific to OC (Additional file 6: Fig. S5A). Moreover, we added new experiments, and our results showed that KIAA1429 knockdown did not affect the level expression of HIF-1a or c-Myc protein, which means that KIAA1429 did not regulate ENO1 through HIF-1a or c-Myc, KIAA1429-ENO1 is a new glycolytic regulatory pathway. It is not related to HIF-1a or c-Myc (Additional file 7: Fig. S6A).

### SPI1 contributed to the transcriptional activation of KIAA1429

**Fig. 7:**
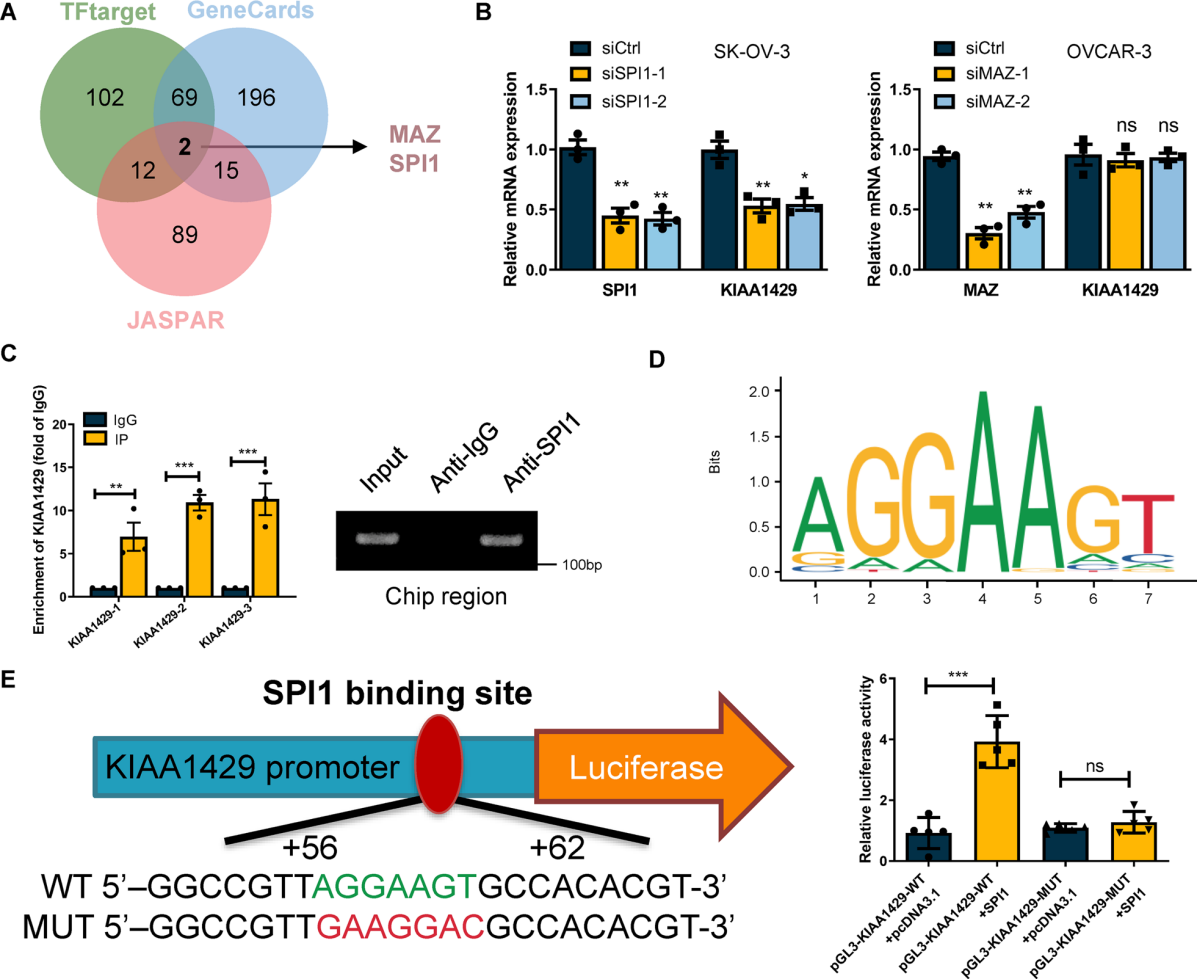
SPI1 promoted KIAA1429 transcription. **A** Venn diagram showing transcription factors that bind the promoter region of KIAA1429 identified in the GeneCards, JASPAR and TFtarget databases. **B** Effect of SPI1 or MAZ knockdown on KIAA1429 expression. *n* = 3 independent experiments. **C** ChIP analysis of SPI1 binding to the KIAA1429 promoter. The input and IgG served as positive and negative controls, respectively. *n* = 3 independent experiments. **D** Schematic of the putative SPI1 binding motif determined using JASPAR. **E** Cells were transfected with KIAA1429-WT or KIAA1429-MUT promoter constructs, and luciferase activity was analysed after transfection. *n* = 3 independent experiments; *P < 0.05, **P < 0.01, and ***P < 0.001

To identify transcription factors that directly regulate the expression of KIAA1429 in OC, using GeneCards, hTF target, and JASPAR, we were able to predict and test upstream transcription factors of KIAA1429 (Fig. 7A). The expression of KIAA1429 in OC cells was shown to be significantly decreased after SPI1 knockdown, as measured by qRT-PCR, while the expression of KIAA1429 did not change significantly after MAZ knockdown (Fig. 7B). Therefore, SPI1 was found to be the primary transcription factor for KIAA1429 for further study. Direct binding of SPI1 to the KIAA1429 enhancer was verified by chromatin immunoprecipitation (ChIP) assay (Fig. 7C). The transcription factor motif analysis of the KIAA1429 promoter sequence was downloaded from the JASPAR database (Fig. 7D). We then established HEK293T cell lines with either WT (AGGAAGT) or MUT (GAAGGAC) SPI1 binding sequences in their respective dual luciferase reporter vectors. Dual luciferase reporter experiment results showed that SPI1 binding site mutation eliminated SPI1's capacity to enhance KIAA1429 transcription (Fig. 7E). Based on our findings, we hypothesize that SPI1 is involved in promoting KIAA1429 transcription via DNA binding motifs (AAGGAAGT).

## Discussion

Eukaryotic mRNAs are typically modified internally by m6A or adenosine N6-methylation [16]. Growing evidence indicates that m6A controls the expression of specific genes involved in cellular processes such as self-renewal, differentiation, invasion, and apoptosis [17, 18]. Installation of m6A modifications is mediated by methyltransferases such as METTL3/14/16, RBM15/15B, ZC3H3, VIRMA, CBLL1, WTAP, and KIAA1429, and is recognized by binding proteins YTHDF1/2/3, YTHDC1/2, IGF2BP1/2/3 and HNRNPA2B [17, 19]. Several studies have reported that the function of m6A methyltransferases in malignancies, as METTL3 accelerates pri-miR221/222 maturation to promote bladder cancer progression [20], ALKBH5 activates PER1 to inhibit pancreatic cancer progression [21]. However, current knowledge of the mechanisms by which m6A modifications affect tumor progression is far from complete.

OC is one of the three major malignant tumors in gynecology. Increasingly evidence has shown that OC is the result of the interaction of genetics, epigenetics, and transcriptomics. Recent studies have also reported that many m6A regulatory factors are abnormally expressed in OC, so methylation of m6A plays an important role in the occurrence and progression of OC [1]. Recently, it was found that YTHDF1 regulates the translation of eIF3C relying on m6A, thus affecting global protein translation in OC, enhancing protein synthesis, and promoting tumorigenesis of OC cells [8]. In addition, high expression of METTL3 has also been reported to be one of the reasons for the abnormal increase in the m6A level, which is related to the malignant degree and survival time of patients with OC [22]. METTL3 gene knockout can significantly inhibit the development and progress of OC cells [23].

As the "writer" of m6A, RNA methyltransferase is an important factor in the abnormal modification of m6A. KIAA1429 is considered the largest m6A methyltransferase and plays an important role in m6A modification. Previous studies have shown that KIAA1429 may play an important role in tumorigenesis [10]. For example, KIAA1429 inhibits ID2 by upregulating m6A modification of ID2 mRNA, thus promoting the migration and invasion of HCC [24]. KIAA1429 regulates the expression of MUC3A by modifying m6A, thus regulating the proliferation and migration of LUAD cells [25]. KIAA1429 can accelerate gefitinib resistance in NSCLC by targeting HOXA1 3'UTR to enhance its mRNA stability [26]. However, the prognostic value and function of KIAA1429 in OC are still unclear. Fan et al. reported that the expression of KIAA1429 was down-regulated in ovarian cancer tissues compared with normal tissues[27], but it was also pointed out that the expression of KIAA1429 in grade III is higher than that in grade II, and patients with higher expression of KIAA1429 had worse prognosis. The results of this investigation showed that the expression of KIAA1429 was significantly higher in OC tissues than in normal tissues. High KIAA1429 levels have been linked to a worse prognosis for OC patients, according to additional analysis using the TCGA database. KIAA1429 overexpression significantly promoted tumor proliferation and reduced apoptosis. In our study, we collected mostly tissue samples from patients with stage III-IV epithelial ovarian cancer and conducted in vitro and in vivo experiments to reach this conclusion. However, it is also important to know that there are many types of ovarian cancer including ovarian epithelial tumors, ovarian malignant germ cell tumors, and ovarian malignant sex cord stromal tumors, etc. Whether the specific expression of KIAA1429 is consistent among different types of ovarian tumors is still unclear, and more in vitro and in vivo experiments are needed to confirm it. In addition, this experiment is limited by the sample size, and all of these may need to be further clarified in future study.

Numerous investigations over the past few decades have established that aerobic glycolysis is a diagnostic feature of cancer [27]. In most instances, aerobic glycolysis is a "selfish" metabolic reprogramming of tumor cells [21]. Aerobic glycolysis is the result of overexpression of hypoxia-inducible factor 1 (HIF-1), activation of the proto-oncogene c-Myc, loss of function of the tumor suppressor p53, activation of PI3K-Akt-mTORC1, Ras, Jak-Stat3 signaling pathways and inactivation of the LKB1-AMPK signaling pathway [28, 29]. Cancer progression depends on the reprogramming of cellular metabolism as a direct consequence of oncogenic mutations [28]. A general characteristic of aerobic glycolysis is the ability to obtain essential nutrients from a nutrient-poor environment and utilize these nutrients for proliferation and metastasis [30, 31]. To date, the role of KIAA1429 in tumor cell metabolic reprogramming has rarely been reported. In the current investigation, we determined for the first time that KIAA1429 plays a crucial role in OC cell glucose metabolism regulation. KIAA1429 overexpression significantly increased glucose absorption and lactate generation and decreased mitochondrial OCR and intracellular pH. This result indicates that KIAA1429 overexpression enables OC cells to produce ATP even under anaerobic conditions, a process that can meet the huge energy demands of OC cells during proliferation and invasion.

RNA-seq analysis combined with metabolomic data identified ENO1 as a downstream target of KIAA1429. During glycolysis, ENO1 is a glycolytic enzyme that triggers the conversion of 2-phosphoglycerate to phosphoenolpyruvate [32, 33]. This oncoprotein has multiple roles, including the deregulation of cellular energy, sustaining tumor development, and blocking apoptosis in cancer cells [34]. Furthermore, ENO1 acts as a plasminogen receptor on the cell surface and promotes cancer invasion and metastasis by angiogenic stimulation [35, 36]. Hsin-Jung Li et al. reported that ENO1 promoted tumor invasion through EGFR and WNT signaling pathway [35]; Tuo Hu et al. reported that ENO1 promoted colon cancer progression by increasing CD47-mediated cell growth and migration [37]. Unfortunately, no research study has previously been conducted to elucidate the role of ENO1 in OC. In our investigation, we described the role of ENO1 in OC, and our findings demonstrated that ENO1 increases proliferation and metastasis. Several previous studies indicated that m6A alteration plays a significant role in mRNA splicing, and when it is disrupted, it results in impaired splicing and subsequent mRNA degradation [5, 38]. At present, there are few discoveries about KIAA1429. We do not deny that KIAA1429 plays a role through other glycolytic enzymes, but in our study, RNA-seq and functional recovery experiments prove that KIAA1429 plays a role mainly by enhancing the stability of ENO1 mRNA. Whether there are other glycolytic enzymes involved in it needs to be further clarified in future experiments. Our results showed that KIAA1429 bound to the ENO1 CDS region and increased ENO1 mRNA stability in an m6A-dependent manner. Functional restoration studies demonstrated that KIAA1429 enhances glycolysis by regulating the mRNA stability of ENO1 in an m6A-dependent manner. Overexpression of ENO1 counteracted the effects of KIAA1429 on tumor growth and metastasis in OC cells.

SPI1 is known as a transcriptional activator to control the on/off status of transcription for several genes involved in glucose metabolism, involving HK2 and PGK1, and is considered a major driver of reprogramming of many glucose metabolisms [39, 40]. SPI1 is overexpressed in a variety of malignancies and has been linked to their ability to proliferate and survive [35]. Baoshun Du et al. reported that SPI1 could promote glioma proliferation and migration [41]; Jianqun Wang et al. reported that SPI1 promoted aerobic glycolysis and cancer progression by reducing neutrophil interaction with cancer cells [35]. The tumorigenicity of SPI1 has been demonstrated in OC and endometrial cancers. Our results demonstrated that SPI1 can bind to and trigger transcription at the KIAA1429 promoter.

**Fig. 8:**
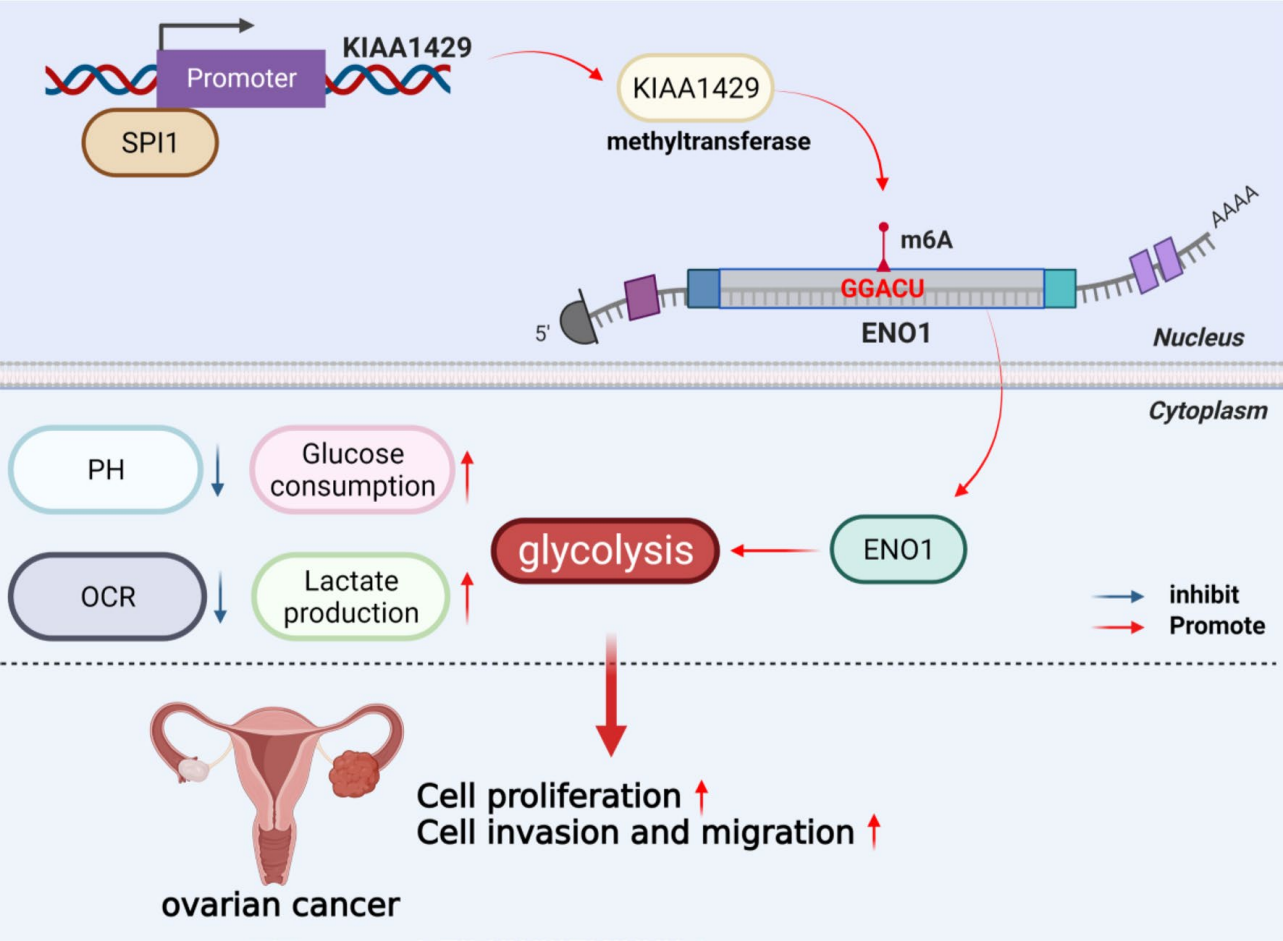
Schematic showing the mechanism of KIAA1429. A schematic model shows that KIAA1429 is transcriptionally activated by SPI1 and regulates ENO1 mRNA stability in an m6A-dependent manner, thereby promoting OC proliferation and metastasis

Our findings revealed a novel regulatory mechanism whereby KIAA1429 is transcriptionally activated by SPI1 and regulates ENO1 mRNA stability in an m6A-dependent manner, thereby promoting OC proliferation and metastasis (Fig. 8). Notably, the regulatory axis of SPI1/KIAA1429/ENO1 in OC has never been reported before. Our conclusions provided unique information on the mechanism of KIAA1429-mediated reprogramming of glucose metabolism in OC progression and paved the way for the future development of OC therapeutic strategies.

### Supplementary Information

**Additional file 1: Table S1. **Primer sequences used in this study. **Table S2. **Primary antibodies used in this study. **Table S3.** siRNAs and shRNA used in this study. **Table S4.** Correlation between clinicopathological features and KIAA1429 expression in OC tumor tissues.

**Additional file 2: Fig. S1.** KIAA1429 overexpression increased OC cell growth and metastasis. **A** qRT-PCR was performed to determine KIAA1429 expression in shRNA control and KIAA1429 knockdown cells. n = 3 independent experiments. **B** qRT-PCR and western blot were performed to determine KIAA1429 expression in Vector and KIAA1429 overexpression cells. n = 3 independent experiments. **C**, **D** Cell proliferation was determined by CCK-8 assay and colony formation assay. n = 3 independent experiments. **E** Cell invasion was determined by Transwell invasion assay. Scale bar, 100μm. n = 3 independent experiments. **F** Cell migration was determined by wound-healing migration assay. Scale bar, 200μm. n = 3 independent experiments; *P < 0.05, **P < 0.01, and ***P < 0.001.

**Additional file 3: Fig. S2.** Regulation of ENO1 mRNA stability by KIAA1429 was m6A-dependent. A Changes in ENO1 mRNA stability after ACT-d treatment. n = 3 independent experiments; *P < 0.05, **P < 0.01, and ***P < 0.001.

**Additional file 4: Fig. S3. **ENO1 promoted OC cell growth and metastasis. **A** qRT-PCR was performed to determine ENO1 expression in siRNA control and ENO1 knockdown cells. n = 3 independent experiments. **B**, **C** CCK-8 and clone formation assays showed that ENO1 functions in cell proliferation. n = 3 independent experiments. **D** Transwell Matrigel invasion assay of cell invasion ability. Scale bar, 50μm. n = 3 independent experiments. **E** Scratch wound-healing motility assay of cell migration. Scale bar, 200μm. n = 3 independent experiments; *P < 0.05, **P < 0.01, and ***P < 0.001.

**Additional file 5: Fig. S4. **KIAA1429 enhanced aerobic glycolysis on OC cells. **A** Glucose uptake, lactate production, pH, and OCR were tested in three different cell lines. **B** The ECAR was measured in three different cell lines using an XF Extracellular Flux Analyzer. n = 3 independent experiments; *P < 0.05, **P < 0.01, ***P < 0.001.

**Additional file 6: Fig. S5. **KIAA1429 is positively correlated with ENO1. A Spearman’s rank correlation analyses showed the correlations between KIAA1429 and ENO1 in TCGA datebase. LIHC: Liver Hepatocellular Carcinoma; PAAD: Pancreatic adenocarcinoma.

**Additional file 7: Fig. S6.** Aerobic glycolysis in OC. **A** Western blot was performed to determine HIF-1a and c-Myc expression in shCtrl and shKIAA1429 cells. n = 3 independent experiments.** B** The results of CCK-8 showed that the proliferation ability of OC cells (SK-OV-3 and OVCAR-3) was significantly reduced in galactose medium. n = 3 independent experiments; **P < 0.01.

## Data Availability

The data that support the findings of this study are available from the corresponding author upon reasonable request.
