# Supplementary material for: N6-methyladenosine methyltransferase KIAA1429 promoted ovarian cancer aerobic glycolysis and progression through enhancing ENO1 expression
Source: Biol Direct. 2023 Oct 9;18:64. doi: 10.1186/s13062-023-00420-7 (PMC10561480; doi:10.1186/s13062-023-00420-7)
Supplement: Supplementary file 1 — Additional file 1: Table S1. Primer sequences used in this study. Table S2. Primary antibodies used in this study. Table S3. siRNAs and shRNA used in this study. Table S4. Correlation between clinicopathological features and KIAA1429 expression in OC tumor tissues. [file 13062_2023_420_MOESM1_ESM.docx]

**Supplementary Table S1** Primer sequences used in this study.

| Primer name | | Sequence (5’-3’) |
| --- | --- | --- |
| KIAA1429 | F | CTTGGCAAGTGGCTTGAACC |
|  | R | ACGTAAGGCAGTGGTAAGGC |
| ENO1 | F | ATGTCTATTCTCAAGATCCATGCCAGG |
|  | R | CTACTTGGCCAAGGGGTTTCTGAAG |
| SPI1 | F | GCCAAACGCACGAGTATT |
|  | R | GCCAAACGCACGAGTATT |
| β-actin | F | CATGTACGTTGCTATCCAGGC |
|  | R | CTCCTTAATGTCACGCACGAT |
| MAZ | F | TGCACAAGCCCTACAACTGCTC |
|  | R | GCACTTGTCTGACGTGACTGTTGA |
| KIAA1429 | F | TACGTGGGCGAGAATTTTCCT |
| Primer 1 | R | TGAGCTTTGGAGCAACGAGA |
| KIAA1429 | F | CAAGGCAGAGTAGAGAAGG |
| Primer 2 | R | GGAGTAATAGAGCAGGATGG |
| KIAA1429 | F | AGGCAGAGTAGAGAAGGATA |
| Primer 3 | R | GGAGTAATAGAGCAGGATGG |

**Supplementary Table S2** Primary antibodies used in this study

| Antigens | Manufacturer | Catalog Number | Application |
| --- | --- | --- | --- |
| KIAA1429 | Proteintech | 25712-1-AP | 1:1000 for WB |
| ENO1 | Proteintech | 11204-1-AP | 1:1000 for WB |
| Fibronectin | Cell Signaling Technology | #26836 | 1:1000 for WB |
| N-cadherin | Cell Signaling Technology | #13116 | 1:1000 for WB |
| E-cadherin | Cell Signaling Technology | #14472 | 1:1000 for WB |
| Vimentin | Cell Signaling Technology | #5741 | 1:1000 for WB |
| MAZ | Proteintech | 21068-1-AP | 1:1000 for WB |
| β-actin | Proteintech | 81115-1-RR | 1:1000 for WB |
| N6-Methylguanosine | Abbkine  Scientific | ABP52775  202003 | Me-RIP  1:500 for Dot Blot |
| IgG | Servicebio | GB23301 | IP |
| HRP | IPKine | A25222 | 1:1000 for WB; IP |

**Supplementary Table S3** siRNAs and shRNA used in this study.

| Primer name | | Sequence (5’-3’) |
| --- | --- | --- |
| sh-NC |  | TTCTCCGAACGTGTCACGT |
| sh-KIAA1429-1 | Sense  Antisense | CACCGCTCAAAGCTGGGACCAAATTCGAAAATTTGGTCCCAGCTTTGAGC  AAAAGCTCAAAGCTGGGACCAAATTTTCGAATTTGGTCCCAGCTTTGAGC |
| sh-KIAA1429-2 | Sense  Antisense | CACCGGAGTTGGTTACCTTGCTTCTCGAAAGAAGCAAGGTAACCAACTCC  AAAAGGAGTTGGTTACCTTGCTTCTTTCGAGAAGCAAGGTAACCAACTCC |
| si-NC |  | UUCUCCGAACGUGUCACGUTT |
| si-ENO1-1 | Sense  Antisense | GCUGCUUACUGUAACUGUAUC  UACAGUUACAGUAAGCAGCUG |
| si-ENO1-2 | Sense  Antisense | GGAGUUGGAGACCAGUCUAGC  UAGACUGGUCUCCAACUCCUG |
| si-MAZ-1 |  | GAGAAGAGAUGGAGUCUUAGG |
| si-MAZ-2 |  | ACAAAUCGUUAAAACCUAGCG |
| si-SPI1-1 | Sense  Antisense | AAGCCAUAGCGAUCACUACUG  CAGUAGUGAUCGCUAUGGCUU |
| si-SPI1-2 | Sense  Antisense | AACAACGAGUUUGAGAACUUC  GAAGUUCUCAAACUCGUUGUU |

**Supplementary Table S4** Correlation between clinicopathological features and KIAA1429 expression in OC tumor tissues.

| Characteristics | Number | KIAA1429 expression | | *P* value |
| --- | --- | --- | --- | --- |
|  |  | High | Low |  |
| Age |  |  |  | 0.168 |
| ≤ 60 | 28 | 12 | 16 |  |
| ＞60 | 12 | 8 | 4 |  |
| Tumor size |  |  |  | 0.376 |
| ≤ 30 mm | 6 | 4 | 2 |  |
| ＞30 mm | 34 | 16 | 18 |  |
| Lymph node metastasis |  |  |  | 0.519 |
| Absent | 16 | 9 | 7 |  |
| Present | 24 | 11 | 13 |  |
| Distant metastasis |  |  |  | ***0.008^*^*** |
| Absent | 14 | 3 | 11 |  |
| Present | 26 | 17 | 9 |  |
| FIGO stage |  |  |  | ***0.013^*^*** |
| I-II | 11 | 2 | 9 |  |
| III-IV | 29 | 18 | 11 |  |
| Pathologic type |  |  |  | 0.058 |
| Serous | 31 | 18 | 13 |  |
| Mucous | 9 | 2 | 7 |  |

*The expression of KIAA1429 was compared between the tumor tissue and the normal tissue. FIGO, Federation International of Gynecology and Obstetrics. *P<0.05
