# Supplementary figures and images for: N6-methyladenosine methyltransferase KIAA1429 promoted ovarian cancer aerobic glycolysis and progression through enhancing ENO1 expression
Source: Biol Direct. 2023 Oct 9;18:64. doi: 10.1186/s13062-023-00420-7 (PMC10561480; doi:10.1186/s13062-023-00420-7)

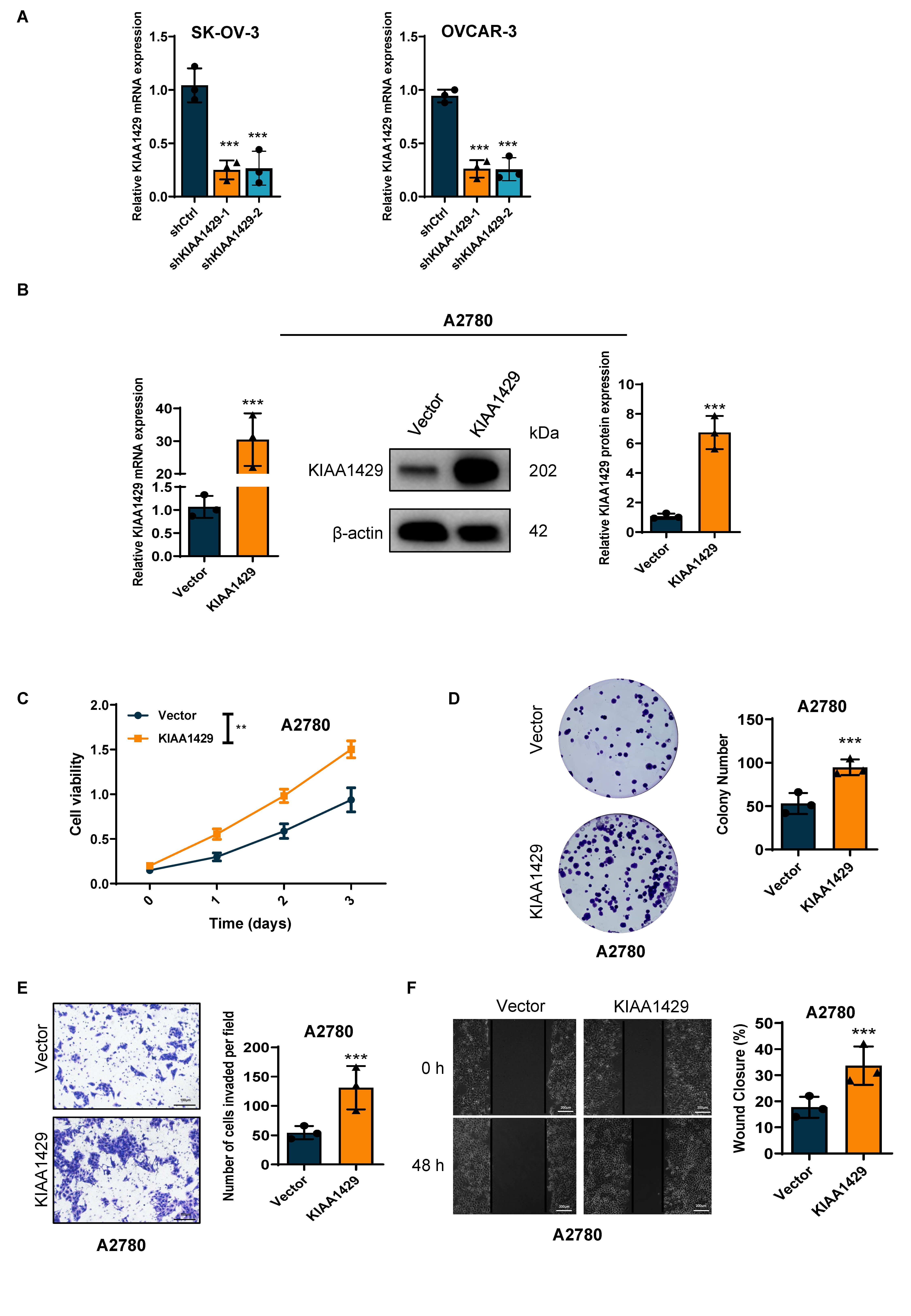

Supplement: Supplementary file 2 — Additional file 2: Fig. S1. KIAA1429 overexpression increased OC cell growth and metastasis. A qRT-PCR was performed to determine KIAA1429 expression in shRNA control and KIAA1429 knockdown cells. n = 3 independent experiments. B qRT-PCR and western blot were performed to determine KIAA1429 expression in Vector and KIAA1429 overexpression cells. n = 3 independent experiments. C, D Cell proliferation was determined by CCK-8 assay and colony formation assay. n = 3 independent experiments. E Cell invasion was determined by Transwell invasion assay. Scale bar, 100μm. n = 3 independent experiments. F Cell migration was determined by wound-healing migration assay. Scale bar, 200μm. n = 3 independent experiments; *P < 0.05, **P < 0.01, and ***P < 0.001. [file 13062_2023_420_MOESM2_ESM.tiff]

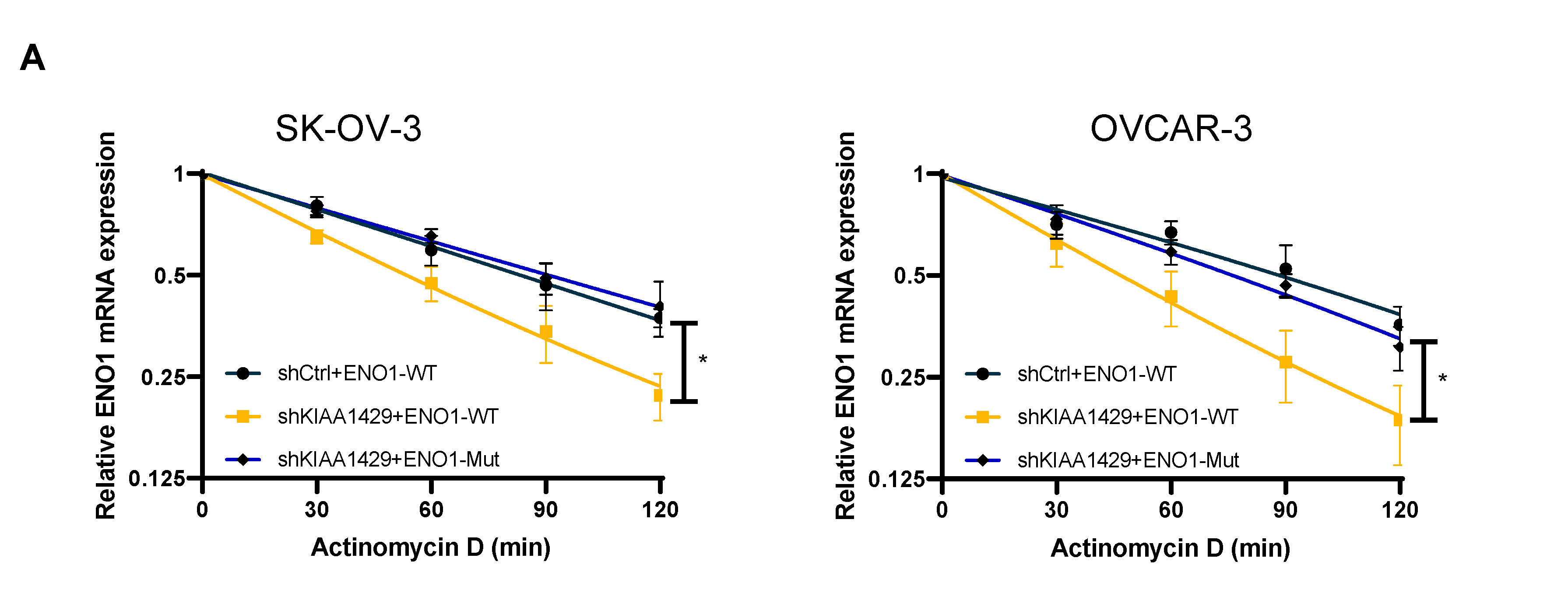

Supplement: Supplementary file 3 — Additional file 3: Fig. S2. Regulation of ENO1 mRNA stability by KIAA1429 was m6A-dependent. A Changes in ENO1 mRNA stability after ACT-d treatment. n = 3 independent experiments; *P < 0.05, **P < 0.01, and ***P < 0.001. [file 13062_2023_420_MOESM3_ESM.tif]

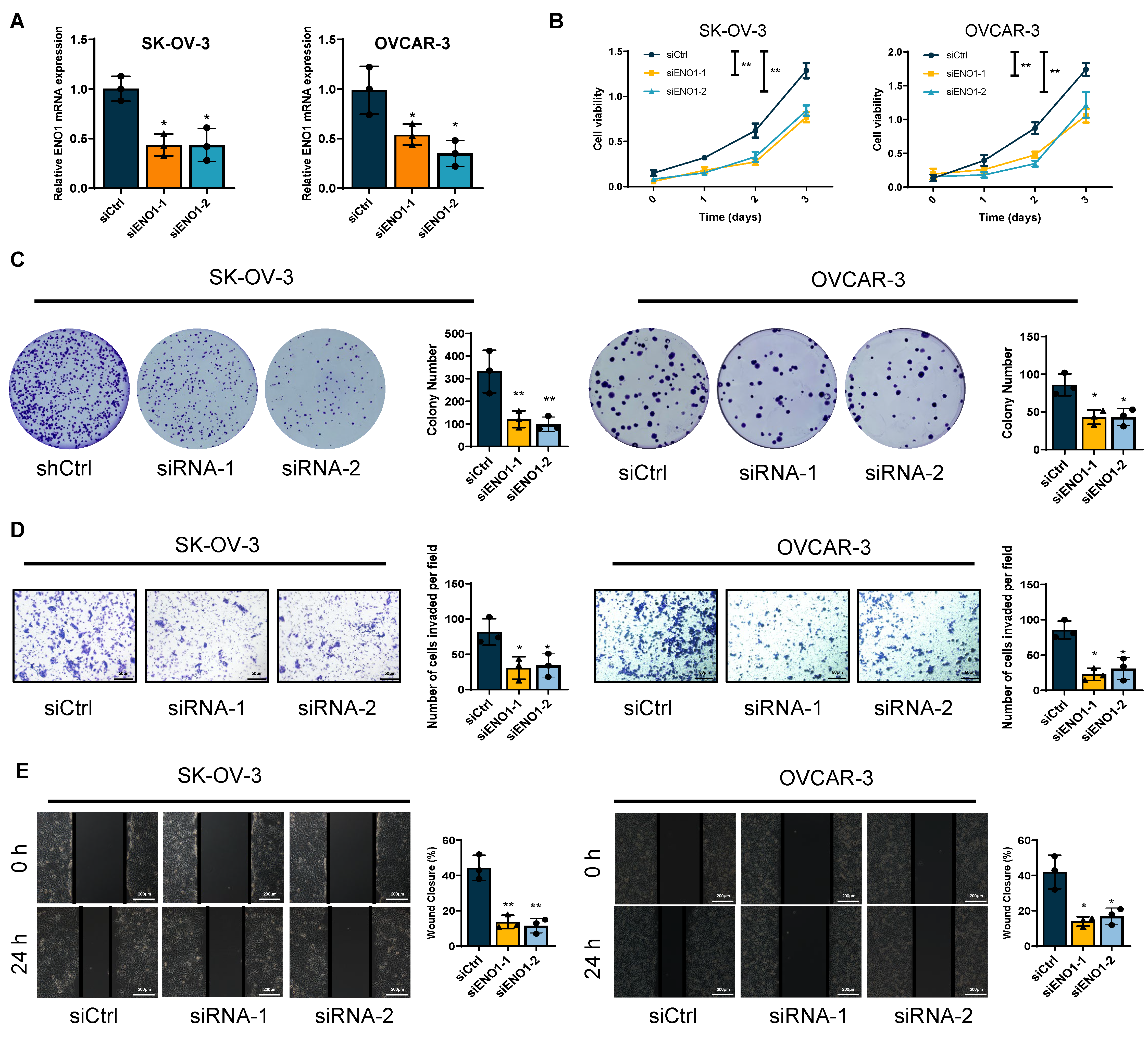

Supplement: Supplementary file 4 — Additional file 4: Fig. S3. ENO1 promoted OC cell growth and metastasis. A qRT-PCR was performed to determine ENO1 expression in siRNA control and ENO1 knockdown cells. n = 3 independent experiments. B, C CCK-8 and clone formation assays showed that ENO1 functions in cell proliferation. n = 3 independent experiments. D Transwell Matrigel invasion assay of cell invasion ability. Scale bar, 50μm. n = 3 independent experiments. E Scratch wound-healing motility assay of cell migration. Scale bar, 200μm. n = 3 independent experiments; *P < 0.05, **P < 0.01, and ***P < 0.001. [file 13062_2023_420_MOESM4_ESM.tiff]

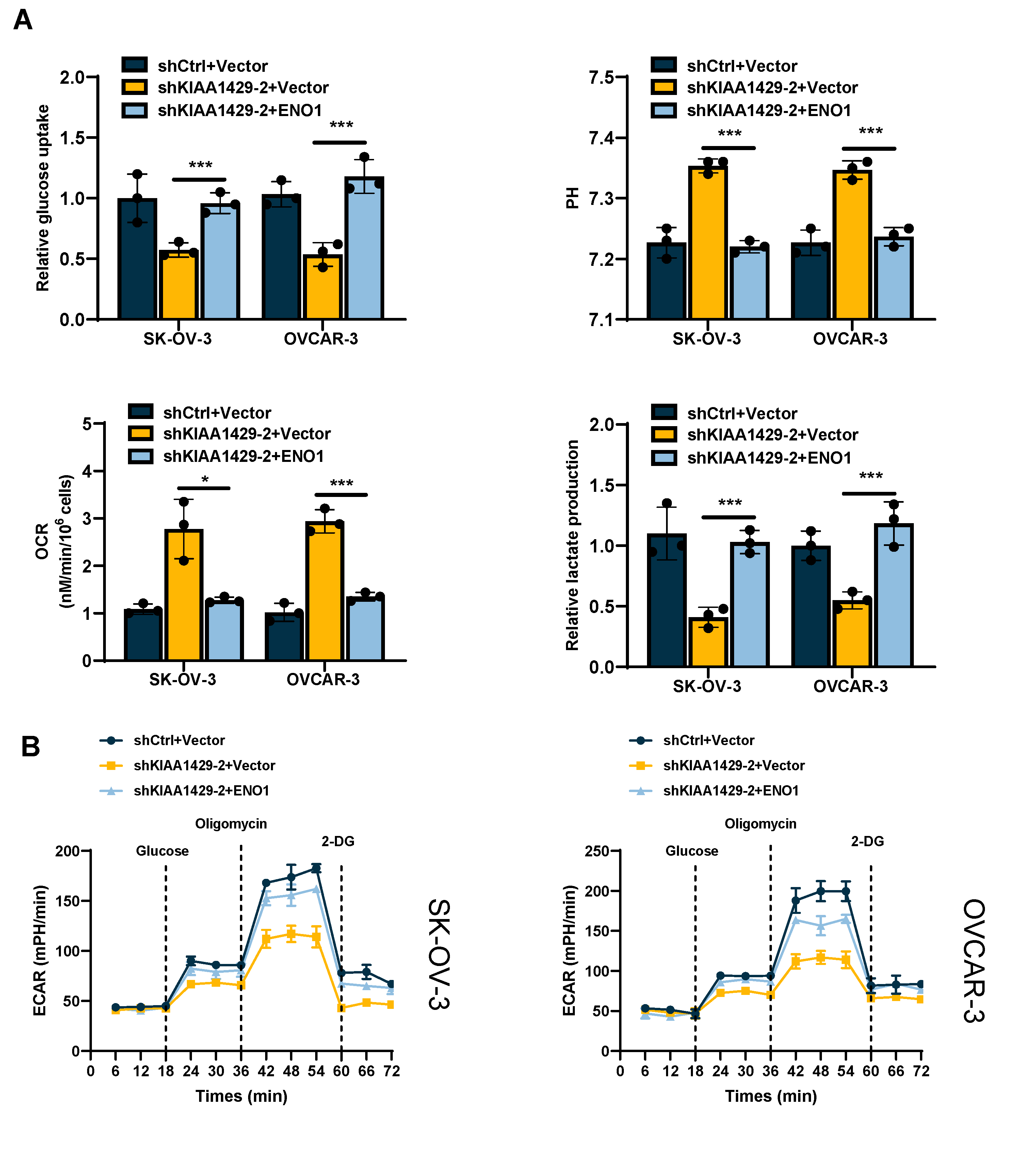

Supplement: Supplementary file 5 — Additional file 5: Fig. S4. KIAA1429 enhanced aerobic glycolysis on OC cells. A Glucose uptake, lactate production, pH, and OCR were tested in three different cell lines. B The ECAR was measured in three different cell lines using an XF Extracellular Flux Analyzer. n = 3 independent experiments; *P < 0.05, **P < 0.01, ***P < 0.001. [file 13062_2023_420_MOESM5_ESM.tiff]

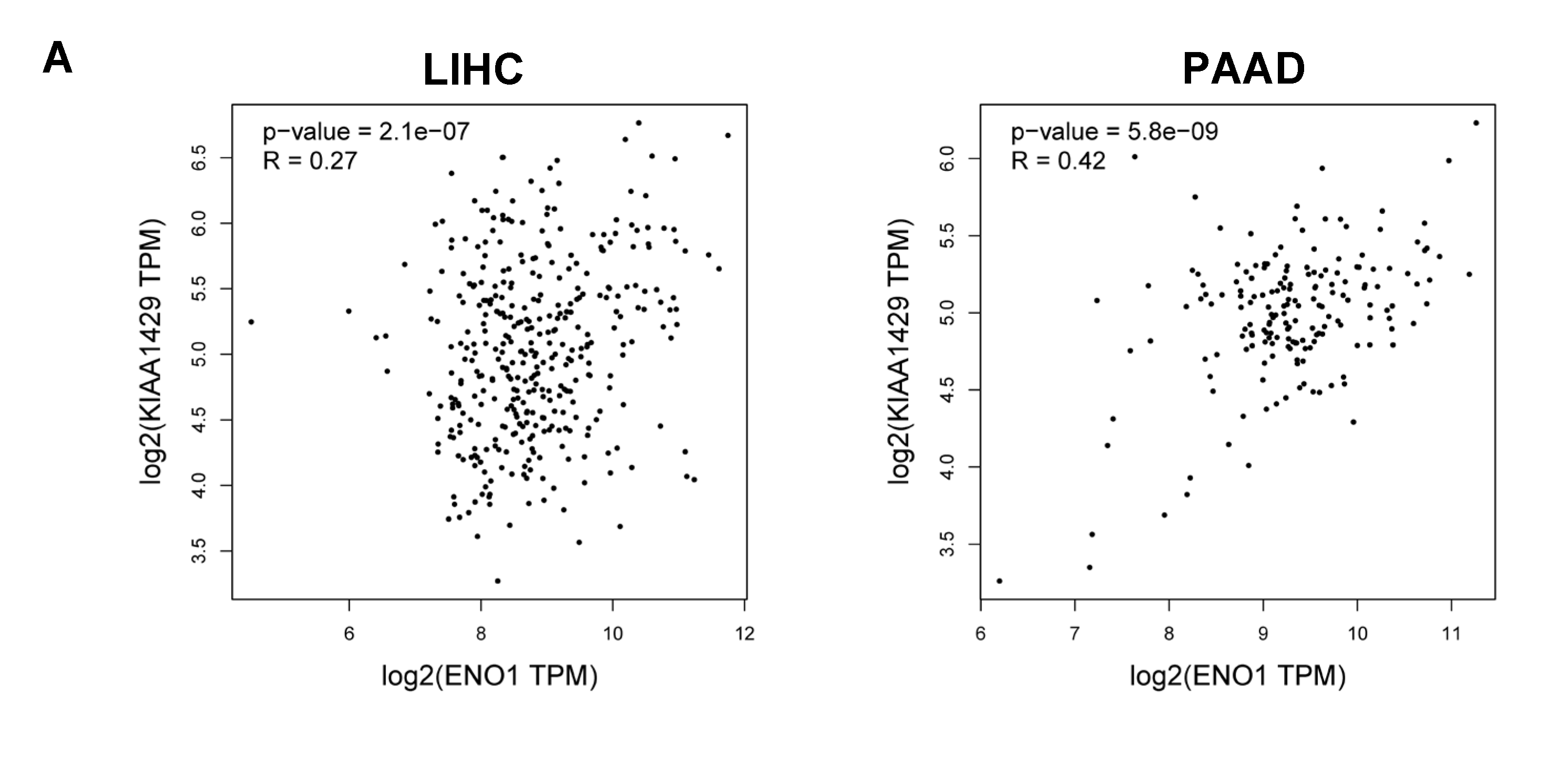

Supplement: Supplementary file 6 — Additional file 6: Fig. S5. KIAA1429 is positively correlated with ENO1. A Spearman’s rank correlation analyses showed the correlations between KIAA1429 and ENO1 in TCGA datebase. LIHC: Liver Hepatocellular Carcinoma; PAAD: Pancreatic adenocarcinoma. [file 13062_2023_420_MOESM6_ESM.tiff]

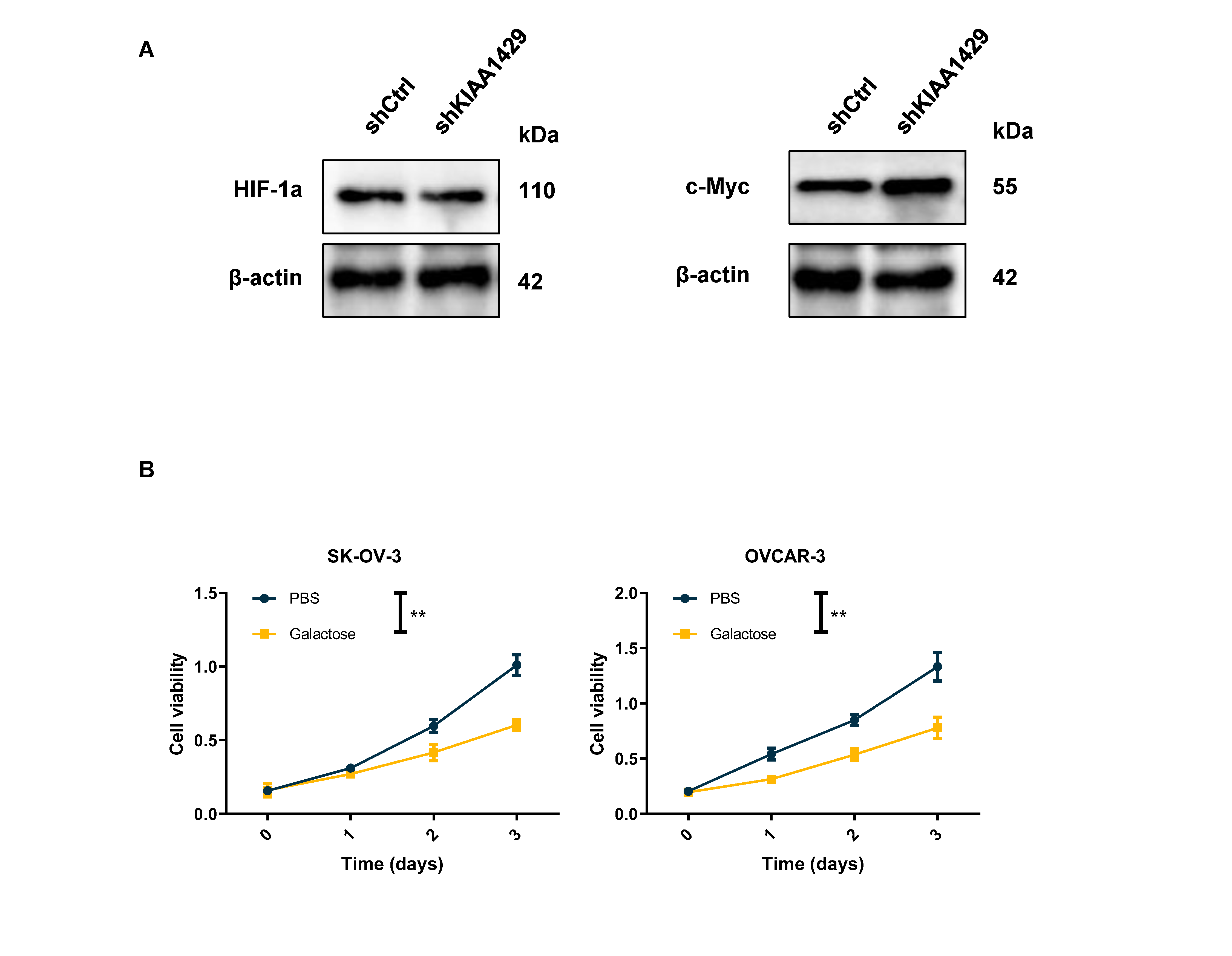

Supplement: Supplementary file 7 — Additional file 7: Fig. S6. Aerobic glycolysis in OC. A Western blot was performed to determine HIF-1a and c-Myc expression in shCtrl and shKIAA1429 cells. n = 3 independent experiments. B The results of CCK-8 showed that the proliferation ability of OC cells (SK-OV-3 and OVCAR-3) was significantly reduced in galactose medium. n = 3 independent experiments; **P < 0.01. [file 13062_2023_420_MOESM7_ESM.tiff]
